# Seasonal variability, antibiogram and genetic diversity of *Vibrio* spp. recovered from effluent discharge of wastewater treatment plants and their receiving rivers in Durban, South Africa

**DOI:** 10.1007/s10661-025-14171-7

**Published:** 2025-06-10

**Authors:** Kerisha Ramessar, Ademola O. Olaniran

**Affiliations:** Discipline of Microbiology, School of Life Sciences, University of KwaZulu-Natal (Westville Campus), Durban, 4000 South Africa

**Keywords:** *Vibrio* spp., Wastewater treatment, Antibiotic resistance, Genetic fingerprinting, Public health

## Abstract

Wastewater treatment plants (WWTPs) play a critical role in mitigating microbial contamination in aquatic environments. However, the persistence of pathogenic bacteria, including *Vibrio* spp., in treated effluents poses a significant public health risk. This study investigates the prevalence, seasonal variation, antibiotic resistance, and genetic diversity of *Vibrio* spp. in effluents from four WWTPs in Durban, South Africa and their receiving rivers. The removal efficiency of *Vibrio* spp. varied significantly across WWTPs, ranging from 76.41 to 100%, with biofilter-based systems, demonstrating the highest efficacy. Molecular identification confirmed *Vibrio vulnificus* and *Vibrio alginolyticus* as dominant species. Antibiotic susceptibility testing revealed widespread resistance, particularly to beta-lactams, with multidrug resistance (*MDR*) detected in 98% of isolates. The multiple antibiotic resistance index (*MARI*) values exceeded 0.2 in 99.5% of isolates, indicating significant antibiotic exposure. Genetic fingerprinting via repetitive extragenic palindromic PCR (REP-PCR) identified identical strains in effluent and downstream river samples, implicating treated effluent as a significant contamination source. These findings underscore the urgent need for improved wastewater treatment processes and stringent regulatory monitoring to mitigate the environmental and public health risks associated with antimicrobial-resistant *Vibrio* spp. Future research should focus on whole-genome sequencing to characterise resistance genes and assess the long-term impact of *Vibrio* contamination on aquatic ecosystems and human health.

**Supplementary Information:**

The online version contains supplementary material available at 10.1007/s10661-025-14171-7.

## Introduction

Wastewater treatment plants (WWTPs) are centralised facilities designed to improve wastewater quality by reducing pollutants to permissible levels before discharging treated effluent into receiving water bodies (Igere et al., 2022). This process is critical due to the potential health risks posed by microbial pollutants, which can significantly impact both public health and environmental integrity (Mbanga et al., 2020). Wastewater typically comprises urban and industrial effluent with a containing a complex mixture of contaminants including nutrients (nitrogen and phosphorus), pathogens (bacteria, protozoa, and viruses), chemicals (pharmaceuticals and personal care products) heavy metals, and antibiotic resistance organisms. Hence, proper treatment of wastewater is essential to comply with regulatory standards and safeguard human and environmental health (Fiorentino et al., 2018, 2019; Kumar et al., 2019; Triggiano et al., 2020; Yang et al., 2019; Zerva et al., 2021).

In South Africa, organisations like the World Health Organisation (WHO) and the Water Research Commission (WRC) have initiated programs to enhance water treatment standards. One significant initiative is the WHO’s “Wastewater and Environmental Surveillance of Pathogens” (WES) program, which monitors pathogens in wastewater to safeguard public health (Osunla et al., 2021). Despite these efforts, the state of WWTPs in South Africa reveals substantial inefficiencies that pose risks to public health and the environment. A 2017 Performance Audit on Water Infrastructure, the South African Department of Water and Sanitation disclosed that 40 to 50% of the country’s 1400 wastewater facilities were in unsatisfactory condition, presenting medium to high risks to the public health (Osunla et al., 2021). The primary causes of these inefficiencies include aging infrastructure, insufficient funding, limited technical expertise, poor management, and non-compliance with wastewater treatment standards (Vaidya et al., 2024).

Urban areas like the eThekwini municipality in Durban are particularly affected by rapid population growth, projected to reach 4.4 million by 2030, which has strained wastewater infrastructure. Informal settlements within this municipality, comprising approximately 312,000 households, often lack adequate sanitation and waste management services, contributing to environmental degradation (eThekwini Municipality, 2022; Sutherland et al., 2014). Addressing these inefficiencies is crucial for safeguarding public health, protecting natural water systems, and ensuring sustainable development (Abbasi et al., 2022).

Monitoring the quality of wastewater released from WWTPs is essential since improperly treated effluent can introduce significant chemical and microbial contamination into freshwater ecosystems (Zerva et al., 2021). Pathogens such as *Escherichia coli*, faecal coliforms, *Bacillus*, *Pseudomonas*, *Clostridium*, *Mycobacterium*, *Aeromonas*, *Vibrio* species, and enteric viruses have been detected in both influent and treated effluents of WWTPs (Abioye et al., 2021; Osunmakinde et al., 2019; Osuolale & Okoh, 2018). For instance, *E. coli* counts in four WWTPs located in uMgungundlovu District, KwaZulu-Natal ranged from 1.1 × 10^5^ CFU/ml in influent to 4.3 × 10^3^ CFU/ml in effluent (Gumede et al., 2021). Similarly, total enterococci counts in municipal WWTPs in Durban was reported to vary significantly between influents (6.1 to 7.2 log₁₀ CFU/100 ml) and effluent samples with 0 and 4.4 log₁₀ CFU/100 ml (Adegoke et al., 2022). Additionally, presumptive *Vibrio* species densities in two Eastern Cape WWTPs and receiving water bodies were reported to range from 1.05 ± 0.23 to 2.08 ± 0.07 log CFU/ml throughout sampling period (Osunla et al., 2021). Such contaminated treated effluents can introduce pathogens responsible for illnesses including diarrhoea, respiratory infections and gastrointestinal diseases in humans into fresh water systems (Zhan et al., 2024).

Among various pathogens present in wastewater effluents, *Vibrio* species are particularly concerning due to their association with severe human diseases such as cholera and gastroenteritis. *Vibrio* species are halophilic Gram-negative bacteria commonly found in marine environments but also present in freshwater systems contaminated by wastewater discharges (Rukawo & Mukaro, 2022; Sampaio et al., 2022). The most prevalent *Vibrio* species identified in treated wastewater effluents include *V*. *cholerae*, *V*. *mimicus*, *V*. *fluvialis*, *V*. *parahaemolyticus* and *V*. *alginolyticus* (Mavhungu et al., 2023; Osunla et al., 2021). *V. vulnificus* is regarded as an opportunistic human pathogen linked to severe infections following exposure through contaminated seafood or water (Conrad & Harwood, 2022; Thompson et al., 2009).

Despite documented occurrences of *Vibrio* species in wastewater systems throughout South Africa’s Eastern Cape Province, where studies have shown high prevalence rates, there remains a significant gap regarding their prevalence specifically within Durban’s WWTPs. This lack of data is concerning given the public health implications associated with these pathogens.

This study aims to evaluate the efficacy of four WWTPs in Durban concerning their ability to eliminate *Vibrio* spp. from incoming influent while analysing the effects of discharged treated effluent on receiving rivers. Additionally, it determines the antibiograms of *Vibrio* spp. recovered from treated wastewater effluent and adjacent surface waters while performing genetic fingerprinting on selected isolates to establish genetic connections among them.

By addressing these critical issues surrounding wastewater treatment efficacy and pathogen prevalence in Durban’s WWTPs, this research contributes valuable insights into public health risks associated with improperly treated wastewater effluents while highlighting the need for improved regulatory compliance and infrastructure investment.

## Materials and methods

### Study area

**Fig. 1:**
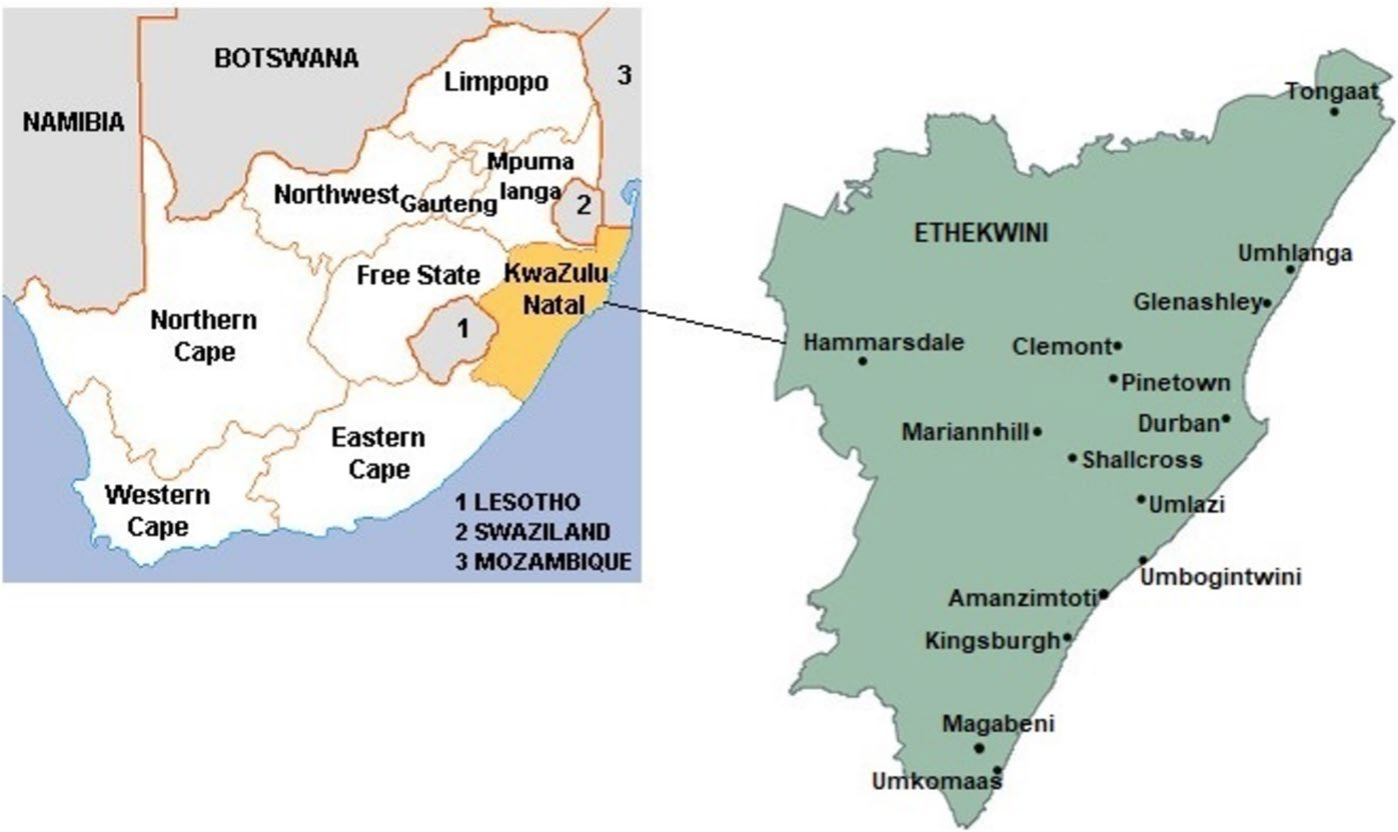
Map of the eThekwini municipality, KwaZulu-Natal (http://www.worldlicenseplates.com/world/AF_ZAKN.html*, *https://municipalities.co.za/map/5/ethekwini-metropolitan-municipality)

The study was conducted in the eThekwini municipality, which extends from its original centre in the port of Durban, KwaZulu-Natal, South Africa (Fig. 1). The geographical coordinates of the four wastewater treatment plants (WWTPs) examined are as follows: WWTP1 at 29°47′43″S 30°59′52″E, WWTP2 at 29°40′43″S 31°02′01″E, WWTP3 at 29°59′25″S 30°54′21″E, and WWTP4 at 29°50′42″S 30°53′27″E. All the wastewater treatment plants discharge their treated effluents directly into rivers. Sample collection was conducted with the authority of the with the eThekwini municipality. WWTP1, WWTP2, and WWTP4 employ activated sludge systems, while WWTP3 utilises biofilter technology for the treatment process.

### Collection of water samples

Seasonal collection of wastewater samples was conducted at various treatment stages from each plant: (1) influent, (2) before to chlorination, (3) activated sludge, (4) post-chlorination, (5) upstream of the receiving river, and (6) downstream of the receiving river. Water samples were gathered in 5-l sterile containers that were rinsed with 70% (v/v) ethanol, followed by deionized water. To maintain sterility, the use of ethanol for sterilizing the 5-l containers was justified by its effectiveness in eliminating microbial contamination. The container’s opening was positioned against the water current to allow water to enter. If no current was present, water samples were obtained using a sampling stick and subsequently transferred into each container. Containers were sealed, stored away from sunlight on ice, and transported to the University of KwaZulu-Natal (Westville campus), where they were maintained at 4 °C. Sample analysis was conducted within 48 h of collection.

### Enumeration of presumptive *Vibrio* spp.

Fifty millilitres of each serially diluted wastewater sample (in sterile distilled water) was filtered using a 0.45-µm cellulose nitrate microfiltration membrane (chm, CHMLAB, Barcelona, Spain). The membrane filter was subsequently positioned on thiosulfate-citrate-bile salts-sucrose agar (TCBS) (Sigma-Aldrich, St. Louis, MO, USA) plates and incubated at 37 °C for 24 h. Green and yellow colonies were counted as putative *Vibrio* isolates and expressed as colony-forming units per millilitre (CFU/ml). The removal efficiency (%) of each indicator microorganism for each wastewater treatment plant (WWTP) was determined using equation: (*B* − *A*)/*B* × 100 (Tao et al., 2010), where *B* denotes the number of presumptive selected indicator microorganisms in the influent, and *A* denotes the number in the treated effluent (post-chlorination). Data were statistically analysed using IBM-SPSS v28.0 (IBM Corp., Armonk, NY, USA). Normality of residuals for each parameter was tested using the Shapiro–Wilk test prior to conducting ANOVA. With all *p*-values above 0.05, normality was confirmed, allowing for appropriate statistical analysis, with means separated using Duncan’s multiple range test at the 5% probability level.

### Isolation and molecular identification of *Vibrio* spp.

Genomic DNA was extracted from the presumptive *Vibrio* spp. using the boiling method as previously outlined (Bai et al., 2010). *Vibrio* species-specific primers (Table 1) were used to conduct PCR in a final volume of 20 µl, comprising 10.1 µl of double-distilled water, 0.02 mM of dNTPs (Thermo-Fisher, Waltham, MA, USA), 1 × PCR buffer, 1.5 mM MgCl_2_, 2.5 U of *Taq* polymerase (JMA, Separation Scientific, South Africa), 1.5 µl of genomic DNA, and varying concentrations of each primer (Inqaba Biotec, Pretoria, South Africa): 0.05 µM for *V16S* forward and reverse primers (Table 1) targeting *Vibrio* species, and 0.25 µM for *V.v.hsp* forward and reverse primers targeting *V. vulnificus* (Tarr et al., 2007). The thermal parameters (T100 Thermal Cycler, Bio-Rad, USA) were adhered to as follows: initial denaturation at 93 °C for 15 min, followed by 35 cycles of denaturation at 92 °C for 40 s, annealing at specified temperatures (Table 1) for 1 min, elongation at 72 °C for 1.5 min, and a final elongation at 72 °C for 7 min.

**Table 1:** Primer sequences and corresponding product sizes for identification of *Vibrio* spp

Target species	Primer	Sequence (5′−3′)			
			*Ta* (℃)	Amplicon size (bp)	References
*V*. *alginolyticus*	*VA16 F1*	ATTGAAGAGTTTGATCATGGCTCAGA	50	1500	Liu et al. (2004)
	*VA16 F2*	CCTTCGGGTTGTAAAGCACT		1000	
	*VA16R2*	TCCTCCCGTAGTTGAAACTACCTACT			
*V*. *vulnificus*	*V.v.hsp −326F*	GTCTTAAAGCGGTTGCTGC	57	410	Tarr et al. (2007)
	*V.v.hsp −697R*	CGCTTCAAGTGCTGGTAGAAG			
All *Vibrio* spp.	*V16S-700 F*	CGGTGAAATGCGTAGAGAT	57	663	Tarr et al. (2007)
	*V16S-1325R*	TTACTAGCGATTCCGAGTTC			
REP-PCR	REP2I	ICGICTTATCIGGCCTAC	40		Versalovic et al. (1994)
	REPIR	IIIICGICGICATCIGGC			

*Ta* annealing temperature

A semi-nested PCR (Liu et al., 2004) was performed to identify isolates as *V. alginolyticus*. The 25-µl reaction mixture comprised 6.25 µl of double distilled water, 0.25 mM dNTP stock, 1 × buffer, 2.5 mM MgCl_2_, 5 U of *Taq* polymerase (250 U), 2 µl of genomic DNA, and 1 µM of each *VA16S* primer (Table 1), targeting the housekeeping genes of *V*. *alginolyticus*. The PCR cycling conditions were 30 cycles of denaturation at 94 °C for 1 min, annealing temperature at 50 °C for 1 min, elongation at 72 °C for 2 min, and a final elongation at 72 °C for 10 min.

The PCR results were visualised using the Chemigenius Bioimaging System (Syngene, Cambridge, UK) following electrophoresis in 1.5% (w/v) agarose (1 h, 100 V) and staining with 1% (w/v) ethidium bromide (15 min). Cultures of *V. parahaemolyticus* (ATCC 17802), *V. vulnificus* (ATCC 27562), and *V. alginolyticus* (ATCC 17749), which were cultured on TCBS, served as positive controls.

### Antibiogram characterisation

Antibiotic susceptibility testing of the isolates were conducted using the Kirby-Bauer disc diffusion method as previously described by Clinical and Laboratory Standards Institute (CLSI) (CLSI, 2015). The isolates were inoculated onto nutrient broth (Merck KGaA, Darmstadt, Germany) and incubated at 37 °C for 24 h, thereafter, standardized to 0.5 McFarland standard and swabbed onto Mueller–Hinton agar plates (Merck KGaA, Darmstadt, Germany). The plates were dried for approximately 30 min prior to placing five antibiotic discs (Oxoid, UK) at equal distances from one another. The 18 antibiotics that were tested against the *Vibrio* isolates comprised ampicillin (10 µg), ampicillin-sulbactam (20 µg), amoxicillin-clavulanate (20/10 µg), penicillin (10 U), amikacin (30 µg), gentamicin (10 µg), cefotaxime (30 µg), cefoxitin (30 µg), ceftazidime (30 µg), choramphenicol (30 µg), nalidixic acid (30 µg), ciprofloxacin (5 µg), ofloxacin (5 µg), deoxycycline (30 µg), tetracycline (30 µg), imipenem (10 µg), trimethoprim-sulfamethoxazole (1.25/23.75 µg), and sulfonamide (300 µg). Each isolate underwent testing in triplicate. The plates were incubated at 37 °C for 24 h and assessed for the presence or absence of clear zones (measured in mm) surrounding the disc, indicating growth inhibition. The isolates were categorized as susceptible, intermediate, or resistant according to the established parameters (CLSI, 2015). Isolates exhibiting resistance to three or more antibiotic classes were considered as multi-drug resistant (Falagas et al., 2006). The quality control strain used was *E. coli* ATCC25922, which was previously cultured in nutrient broth.

### Repetitive extragenic palindromic PCR

Repetitive extragenic palindromic PCR (REP-PCR) was performed using REP2I and REPIR primers (Table 1), as described by (Versalovic et al., 1994). Genomic DNA was isolated using previously outlined methods. The PCR reaction was prepared in a total volume of 25 µl and comprised 9.8 µl of double distilled water, 0.2 mM of dNTPs (Thermo Scientific USA), 1.5 mM of MgCl2, 1 × of PCR buffer, 0.6 µm of each primer (Inqaba Biotec, Pretoria, South Africa), 2 U of Taq polymerase (Supertherm) and 3 µl of template DNA. The PCR protocol included an initial denaturation at 95 °C for 7 min, 30 cycles of 90 °C at 30 s, 40 °C for 1 min, 65 °C for 8 min and a final elongation at 65 °C for 16 min. The resulting amplicons were separated on a 1.5% agarose gel electrophoresed at 80 V for 3 h. The gel was stained with 1% ethidium bromide and visualised under a UV transilluminator using the Chemigenius Bioimaging System (Syngiene). A 1 kb DNA marker (Thermo Scientific, USA) was used as the molecular size standard. Two reference strains (*V. vulnificus*: ATCC 27562 and *V. alginolyticus*: ATCC 17749), which were previously cultured on TCBS, were included in each experiment to validate both PCR amplification and the gel electrophoresis profiles.

### Statistical clustering approaches and dendrogram similarity thresholds

Banding patterns and cluster analysis were conducted using BioNumerics software v.6.6 (Applied Maths and Scientific Software Development, Saint-Martens-Latem, Belgium). Pairwise similarity values were calculated using the Dice coefficient, and dendrograms were constructed using unweighted pair group method with arithmetic mean (UPGMA) clustering. Dendrogram similarity thresholds were adjusted to determine the level of similarity at which clusters were formed. This adjustment allowed for the merging or separation of clusters based on the desired level of similarity, facilitating the effective interpretation of genetic relationships and diversity among the samples analysed.

## Results

### Seasonal variability effects on the *Vibrio* counts (log_10_ CFU/100 ml) of each wastewater treatment plant (WWTP) across the sampling points

The effects of seasonal variations, sampling points (*SP*), and their interaction on *Vibrio* counts across the WWTPs are shown in Table 2. The analysis of variance (ANOVA) revealed that the *Vibrio* counts across the WWTPs were significantly affected by seasonal variations and sampling points (*SP*) across all WWTPs (*p* ≤ 0.001). In WWTP1, *Vibrio* counts showed significant variation across all factors, with season exhibiting a mean square value of 9.06 log_10_ CFU/100 ml, SP (6.01 log_10_ CFU/100 ml), and the interaction of season × *SP* (2.72 log_10_ CFU/100 ml). For WWTP2, both season (1.54 log_10_ CFU/100 ml) and *SP* (9.35 log_10_ CFU/100 ml) had significant effects, while the interaction between season × *SP* contributed minimally (0.5 log_10_ CFU/100 ml). WWTP3 demonstrated a mean square value of 2.71 for season and a particularly high value for *SP* (84.08 log_10_ CFU/100 ml), indicating substantial spatial differences in *Vibrio* counts. Similarly, WWTP4 exhibited significant variation, with mean square values of 4.12 log_10_ CFU/100 ml for season, 12.99 log_10_ CFU/100 ml for *SP*, and 1.53 log_10_ CFU/100 ml for their interaction, highlighting the influence of both seasons and spatial factors.

**Table 2:** ANOVA mean squares effects of seasonal variations on the *Vibrio* counts (log_10_ CFU/100 ml) across the different sampling points of the WWTPs

	*df*	WWTP1	WWTP2	WWTP3	WWTP4
Season	3	9.06*	1.54*	2.71*	4.12*
*SP*	5	6.01*	9.35*	84.08*	12.99*
Season × *SP*	15	2.72*	0.50*	2.22*	1.53*
Error	48	0.02*	0.00*	0.00*	0.00*
Total	72				
Corrected total	71				

*Significantly different at *p* ≤ 0.001

*df* degree of freedom, *WWTP* wastewater treatment plant, *SP* sampling point

### Mean effects of seasonal changes on the *Vibrio* count (log_10_CFU/100 ml) of different WWTPs

The seasonal variation in *Vibrio* counts across the different WWTPs is presented in Table 3. Significant differences were observed across all seasons in the WWTPs, with the exception of autumn and winter in WWTP2, where no significant difference was observed (*p* > 0.05). In WWTP1, the highest mean *Vibrio* count was observed in autumn (6.63 log_10_ CFU/100 ml), while the lowest was recorded in summer (5.05 log_10_ CFU/100 ml). In contrast, WWTP2 showed the highest values in spring (6.20 log_10_ CFU/100 ml) significantly higher than the other seasons. Similarly, WWTP3 exhibited the highest mean in spring (5.29 log_10_ CFU/100 ml) and the lowest in winter (4.41 log_10_ CFU/100 ml). Lastly, in WWTP4, the highest value was observed in summer (6.11 log_10_ CFU/100 ml), while autumn recorded the lowest mean (5.08 log_10_ CFU/100 ml).

**Table 3:** Mean effects of different seasons on concentration of *Vibrio* (log_10_ CFU/100 ml) in the different WWTPs

Season	WWTP1	WWTP2	WWTP3	WWTP4
Spring	6.33	6.20	5.29	5.19
Summer	5.05	5.51	4.58	6.11
Autumn	6.63	5.98 ^NS^	4.68	5.08
Winter	5.61	6.01 ^NS^	4.41	5.72

Mean values with NS superscripted in the same column are not significantly different *p* ≤ 0.05

*WWTP* wastewater treatment plant

### Mean performance of different sampling points on *Vibrio* counts (log_10_ CFU/100 ml) in the different WWTPs

The mean performance of sampling points (*SP*) on *Vibrio* counts across the four WWTPs is presented in Table 4. Significant differences were observed across all *SP*s within each treatment plant, except for the before chlorination (*BC*) and downstream (*DS*) in WWTP1. In WWTP1, the influent (*INF*) recorded the highest mean *Vibrio* count at 6.81 log_10_ CFU/100 ml, while the lowest count was observed in the after chlorination (*AC*) sample at 4.75 log_10_ CFU/100 ml indicating a substantial reduction in *Vibrio* levels post-treatment. In WWTP2, activated sludge (*AS*) had the highest mean count at 6.82 log_10_ CFU/100 ml, reflecting elevated *Vibrio* levels during this stage of treatment, while the lowest count was found in the upstream (*US*) sample at 4.57 log_10_ CFU/100 ml, highlighting lower levels of *Vibrio* present in environmental waters. In WWTP3, the *INF* sample exhibited the highest mean *Vibrio* count at 7.36 log_10_ CFU/100 ml, whereas the *AC* sample recorded the lowest at 3.84 log_10_ CFU/100 ml, suggesting effective treatment. The biofilter stage also showed a reduction in *Vibrio* levels, with a mean value of 5.70 log_10_ CFU/100 ml, indicating its contribution to the treatment process. Similarly, in WWTP4, the highest *Vibrio* counts were observed in *AS* (6.49 log_10_ CFU/100 ml), while the lowest were recorded in *US* (4.21 log_10_ CFU/100 ml).

**Table 4:** Mean performance of different sampling points on *Vibrio* counts (log_10_ CFU/100 ml) in the different WWTPs

*SP*	WWTP1	WWTP2	WWTP3	WWTP4
*INF*	6.81	6.75	7.36	6.44
*BC*	6.07^NS^	6.35	6.73	6.39
*AS*/*BF*	6.22	6.82	5.70	6.49
*AC*	4.75	5.64	3.84	4.54
*US*	5.48	4.57	-	4.21
*DS*	6.09^NS^	5.39	4.82	5.09

Mean values with NS superscripted in the same column are not significantly different *p* ≤ 0.05

*SP* sampling point, *INF* influent, *BC* before chlorination, *AS* activated sludge, *AC* after chlorination, *BF* biofilter, *DS* downstream, *US* upstream

### Removal efficiencies of the WWTPs

**Fig. 2:**
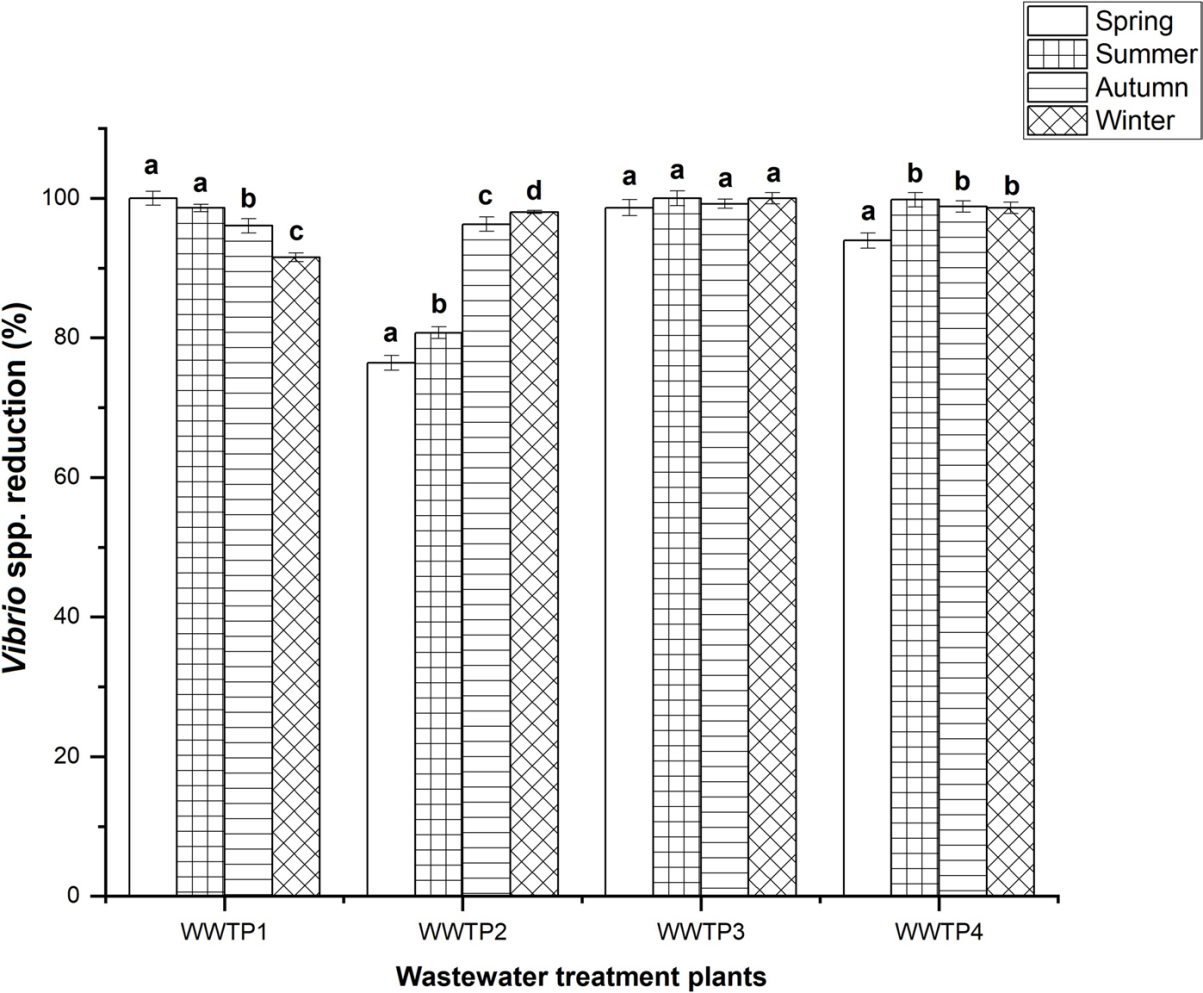
Removal efficiencies of *Vibrio* spp. during treatment processes at the different WWTPs across the seasons. Identical alphabet indicates no significant difference between seasons, whereas different alphabets indicate significant difference between seasons within a plant. Error bars represent the standard deviation of removal

The removal efficiency of *Vibrio* spp. across all seasons varied from 91.52 to 99.99% for WWTP1, 76.41–98.04% for WWTP2, 98.66–100% for WWTP3 and 93.94–99.79% for WWTP4 (Fig. 2). In WWTP1, the highest removal efficiency was observed in spring (99.99%), while winter exhibited the lowest removal efficiency (91.52%). In WWTP2 and WWTP3, removal efficiencies were maximal during winter at 98.04% and 100%, respectively, whereas spring recorded the lowest efficiencies at 76.41% and 98.66%, respectively. For WWTP4, the removal efficiency of 99.79% was highest during summer, whereas spring demonstrated the lowest efficiency of 93.94%.

An analysis of variance was conducted on the removal efficiency of each wastewater treatment plant across different seasons. For WWTP1, the results indicated a significant difference (*p* ≤ 0.05) across all seasons, except between spring and summer. In WWTP2, a significant difference (*p* ≤ 0.05) was observed across all seasons. WWTP3 exhibited no significant differences between any seasons, while a significant difference in the removal efficiency was observed between spring and the other seasons for WWTP4, although no significant differences were found among the latter three seasons.

### Molecular confirmation of *Vibrio* genus, *V*. *vulnificus* and *V*. *alginolyticus*

**Fig. 3:**
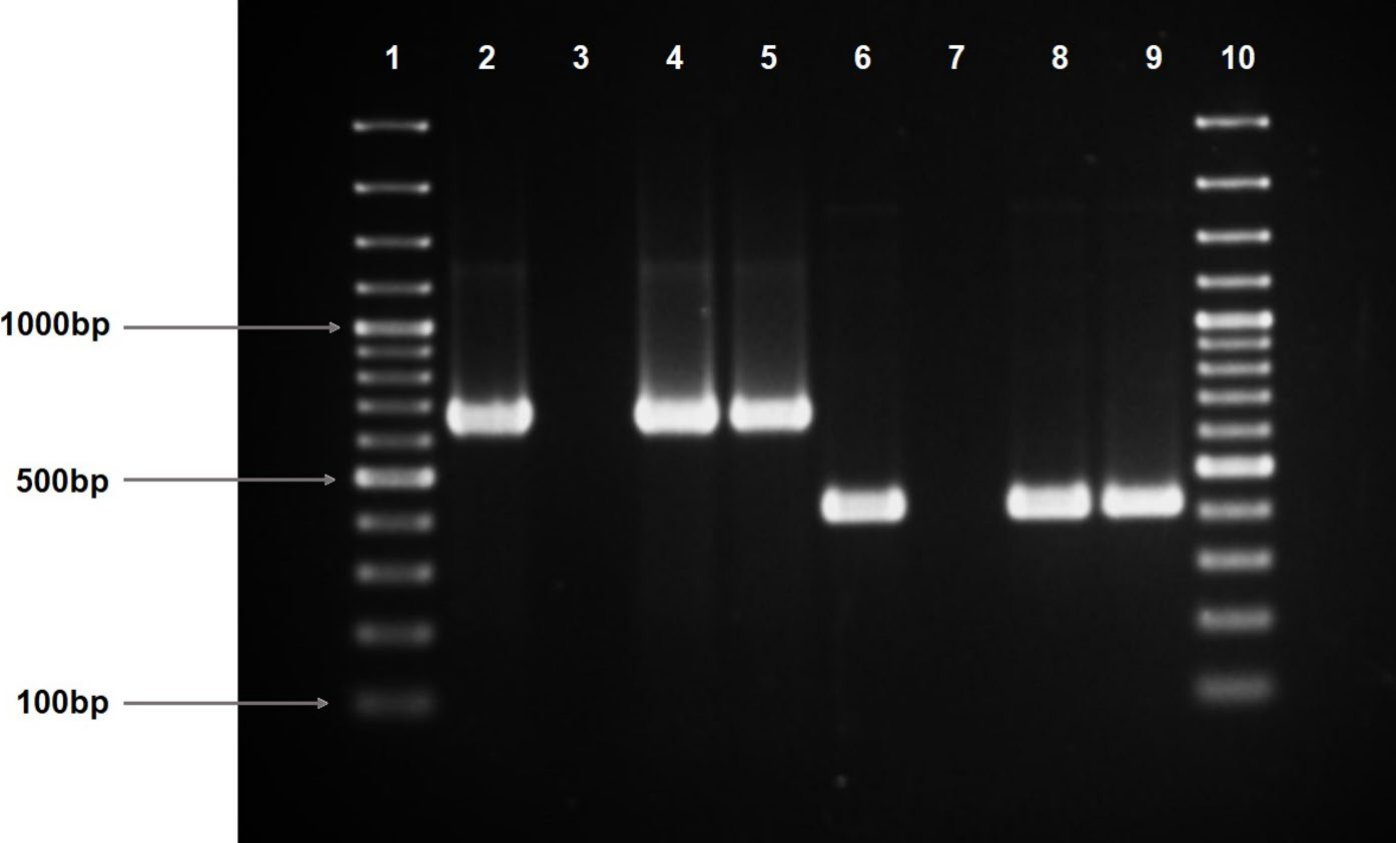
Representative gel of amplified *V16S* gene (663 bp) for *Vibrio* genus identification and *V.v.hsp* (410 bp) for identification of *Vibrio vulnificus* in isolates. Lanes 1 and 10 show the 100 bp molecular marker. Lane 2 shows the PCR product for *V16 s* gene in the positive control (663 bp); lane 3 shows the negative control; and lanes 4 and 5 show the PCR product for *V16S* gene in the representative isolates (663 bp). Lane 6 shows the PCR product for the *V.v.hsp* gene in the positive control (410 bp); lane 7 shows the negative control; and lanes 8 and 9 show the PCR product for *V.v.hsp* gene in the representative isolates (410 bp)

**Fig. 4:**
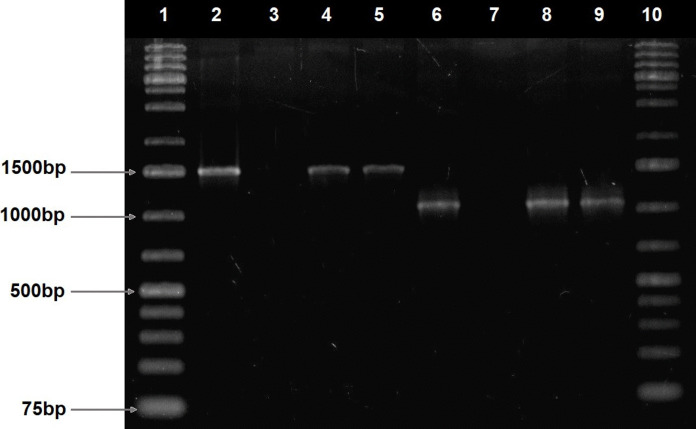
Representative gel showing semi-nested PCR products for *VA16 F1* (1500 bp) and *VA16 F2* (1000 bp) for identification of *Vibrio alginolyticus*. Lanes 1 and 10 show the 1 kb molecular marker. Lane 2 shows the PCR product for *VA16 F1* for the positive control (1500 bp); lane 3 shows the negative control; and lanes 4 and 5 show the PCR product for *VA16 F1* in the representative isolates (1500 bp). Lane 6 shows the PCR product for *VA16 F2* for the positive control (1000 bp); lane 7 shows the negative control; and lanes 8 and 9 show the PCR product for *VA16 F2* in the representative isolates (1000 bp)

Presumptive *Vibrio* isolates were obtained from the discharge point of the treated effluent, as well as from upstream and downstream locations of the receiving surface waters of four wastewater treatment plants in Durban, KwaZulu-Natal. Presumptive *Vibrio* species were isolated and purified on TCBS. Among the purified presumptive *Vibrio* isolates, 200 were verified as *Vibrio* spp. using PCR amplification of the *Vibrio* 16S rRNA gene (663 bp) (Fig. 3; Fig. S1). All isolates underwent molecular analysis to further delineate their pathotypes. Of the 200 isolates, 89% (*n* = 178) were identified as *V. vulnificus*, using PCR amplification of the *V.v.hsp* gene (Fig. 3; Fig. S1), whereas 7.5% (*n* = 15) were identified as *V. alginolyticus* through amplification of the *VA16S* (Fig. 4; Fig. S2), using a semi-nested PCR method. The remaining 3.5% (*n* = 7) of isolates were identified as *Vibrio* spp.

### Antibiogram analysis of *Vibrio* isolates

The antibiotic resistance profile of *Vibrio* spp. recovers at the different sampling points of the wastewater treatment plants, and the receiving rivers investigated in this study are shown in Table 5. Of the 178 V*. vulnificus* strains, 94.4%, 76.4%, 92.7% and 100% of the isolates exhibited resistance to ampicillin, ampicillin-sulbactam, amoxicillin clavulanate and penicillin, respectively. All *V. alginolyticus* isolates exhibited resistance to ampicillin and penicillin, with 46.7% demonstrating resistance to ampicillin-sulbactam and 86.7% to amoxicillin clavulanate. All *Vibrio* spp. isolates exhibited resistance to ampicillin, ampicillin-sulbactam, amoxicillin-clavulanate and penicillin. Resistance rates recorded for tetracycline and doxycycline were 75.3% and 75.8% in *V. vulnificus*, 66.7% and 60% in *V. alginolyticus*, and 85.7% and 100% in *Vibrio* spp., respectively. Resistance was also noted against trimethoprim-sulfamethoxazole and sulfonamide in *V. vulnificus* (61.8% and 91.6%, respectively), in *V. alginolyticus* (73.3% and 100%, respectively) and in *Vibrio* spp. (71.4% and 85.7%). Resistance to imipenem was reported in 69.1% of *V. vulnificus*, 80% of *V. alginolyticus* and 100% of *Vibrio* spp. More than 50% of *V. vulnificus* and *V. alginolyticus* isolates exhibited susceptibility to amikacin and gentamicin; however, 71.4% of *Vibrio* spp. demonstrated susceptibility to gentamicin, while 57.7% exhibited intermediate resistance to amikacin. More than 50% of all *V. vulnificus* and *Vibrio* spp. isolates exhibited susceptibility to ciprofloxacin and ofloxacin, except for *V. alginolyticus*, where only 46.7% of the isolates were susceptible to ciprofloxacin.

**Table 5:** Antibiograms of *Vibrio* isolates (*n* = 200)

Antibiotic class	Antibiotic (µg)	*V. vulnificus* (*n* = 178)			*V. alginolyticus* (*n* = 15)			*Vibrio* spp. (*n* = 7)		
		*S*	*I*	*R*	*S*	*I*	*R*	*S*	*I*	*R*
Beta-lactams	AMP10	2 (1.1)	8 (4.5)	168 (94.4)	0 (0)	0 (0)	15 (100)	0 (0)	0 (0)	7 (100)
	SAM20	20 (11.2)	22 (12.4)	136 (76.4)	3 (20)	5 (33.3)	7 (46.7)	0 (0)	0 (0)	7 (100)
	AMC30	7 (3.9)	6 (3.4)	165 (92.7)	0 (0)	2 (13.3)	13 (86.7)	0 (0)	0 (0)	7 (100)
	P10	0 (0)	0 (0)	178 (100)	0 (0)	0 (0)	15 (100)	0 (0)	0 (0)	7 (100)
Amino-glycosides	AK30	101 (56.7)	49 (27.5)	28 (15.7)	8 (53.3)	3 (20)	4 (26.7)	2 (28.6)	4 (57.1)	1 (14.3)
	CN10	129 (72.5)	31 (17.4)	18 (10.1)	10 (66.7)	3 (20)	2 (13.3)	5 (71.4)	1 (14.3)	1 (14.3)
Cephalosporin	CTX30	47 (26.4)	39 (21.5)	92 (51.7)	0 (0)	3 (20)	12 (80)	0 (0)	1 (14.3)	6 (85.7)
	FOX30	47 (26.4)	40 (22.5)	91 (51.1)	1 (6.7)	0 (0)	14 (93.3)	1 (14.3)	1 (14.3)	5 (71.4)
	CAZ30	95 (53.3)	38 (21.3)	45 (25.3)	5 (33.3)	6 (40)	4 (26.7)	1 (14.3)	2 (28.6)	4 (57.1)
Phenicols	C30	86 (48.3)	50 (28.1)	42 (23.6)	10 (66.7)	4 (26.7)	1 (6.7)	3 (42.9)	4 (57.1)	0 (0)
Quinolone	NA30	67 (37.6)	44 (24.7)	67 (37.6)	3 (20)	5 (33.3)	7 (46.7)	4 (57.1)	1 (14.3)	2 (28.6)
Fluoro-quinolones	CIP5	104 (58.4)	47 (26.4)	27 (15.2)	7 (46.7)	4 (26.7)	4 (26.7)	4 (57.1)	1 (14.3)	2 (28.6)
	OFX5	122 (68.5)	32 (18)	24 (13.5)	8 (53.3)	4 (26.7)	3 (20)	5 (71.4)	1 (14.3)	1 (14.3)
Tetracyclines	DO30	34 (19.1)	10 (5.6)	134 (75.3)	3 (20)	2 (13.3)	10 (66.7)	0 (0)	1 (14.3)	6 (85.7)
	TE30	35 (19.7)	8 (4.5)	135 (75.8)	4 (26.7)	2 (13.3)	9 (60)	0 (0)	0 (0)	7 (100)
Carbapenems	IPM10	11 (6.2)	44 (24.7)	123 (69.1)	0 (0)	3 (20)	12 (80)	0 (0)	0 (0)	7 (100)
Sulfonamides	SXT25	47 (26.4)	21 (11.8)	110 (61.8)	4 (26.7)	0 (0)	11 (73.3)	1 (14.3)	1 (14.3)	5 (71.4)
	S300	9 (5.1)	6 (3.4)	163 (91.6)	0 (0)	0 (0)	15 (100)	1 (14.3)	0 (0)	6 (85.7)

*S* susceptible, *I* intermediate resistance, *R* resistant, *AMP10* ampicillin (10 µg), *SAM20* ampicillin-sulbactam (20 µg), *AMC30* amoxicillin-clavulanate (20/10 µg), *P10* penicillin (10 U), *AK30* amikacin (30 µg), *CN10* gentamicin (10 µg), *CTX30* cefotaxime (30 µg), *FOX30* cefoxitin (30 µg), *CAZ30* ceftazidime (30 µg), *C30* choramphenicol (30 µg), *NA30* nalidixic acid (30 µg), *CIP5* ciprofloxacin (5 µg), *OFX5* ofloxacin (5 µg), *DO30* doxycycline (30 µg), *TE30* tetracycline (30 µg), *IPM10* imipenem (10 µg), *SXT25* trimethoprim-sulfamethoxazole (1.25/23.75 µg), *S300* sulfonamide (300 µg)

### Antibiograms and multiple antibiotic resistance index in isolates

Table S1 presents the association between antibiotic resistance phenotypes and the multiple antibiotic resistance index (*MARI*) among *V. vulnificus*, *V. alginolyticus* and *Vibrio* spp. The *MARI* varied from 0.11 to 1, 022 to 0.89 and 0.56 to 0.83 for *V. vulnificus*, *V. alginolyticus Vibrio* spp., respectively. All *V. alginolyticus* and *Vibrio* spp. demonstrated a multidrug resistant (*MDR*) phenotype, with 97.7% of *V. vulnificus* also exhibiting MDR. One isolate exhibited phenotypic resistance to all tested antibiotics, with *MARI* of 1, followed by an *ARI* of 0.94 demonstrated by two isolates. In *V. vulnificus*, 136 antibiotic resistance patterns were identified, with 107 represented by individual isolates. A total of 12 antibiotic resistance patterns were identified in *V. alginolyticus*, with 11 of these represented by individual isolates and 7 isolates corresponding to the 7 antibiotic resistance patterns observed in *Vibrio* spp. The *V. vulnificus* phenotypes A38 and A50 exhibited the greatest number of isolates (*n* = 5) within a single phenotype, followed by phenotypes A45, A50 and B11 (*n* = 4), as well as phenotype B8 in *V. alginolyticus* (*n* = 4). Phenotypes A30 and B8 were prevalent in *V. vulnificus* and *V. alginolyticus*, phenotypes A38, A39 and A50 were prevalent in *V. vulnificus* and *Vibrio* spp., while phenotype A64 was prevalent across all three species.

### Genetic fingerprinting of *Vibrio* isolates

**Fig. 5:**
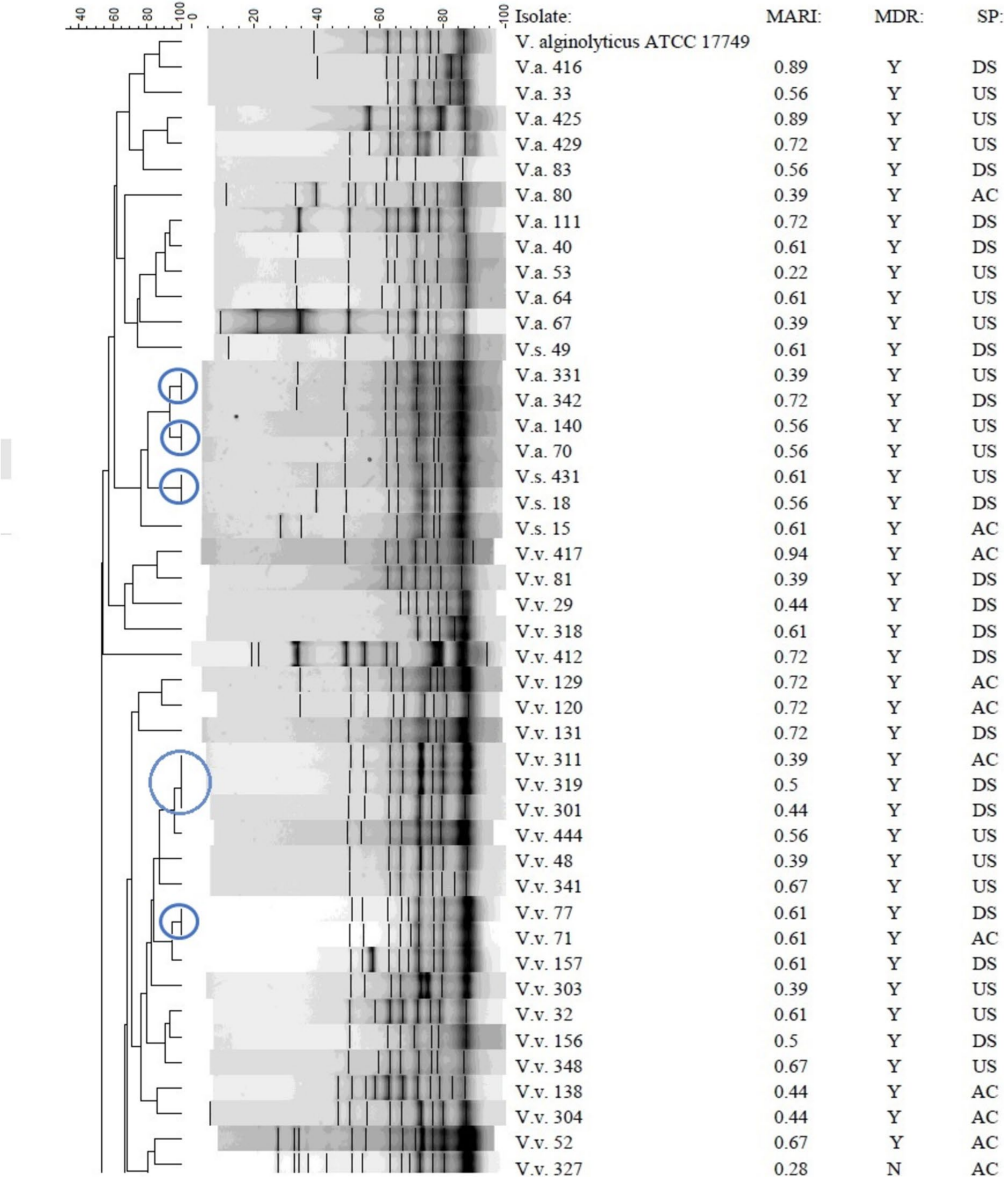

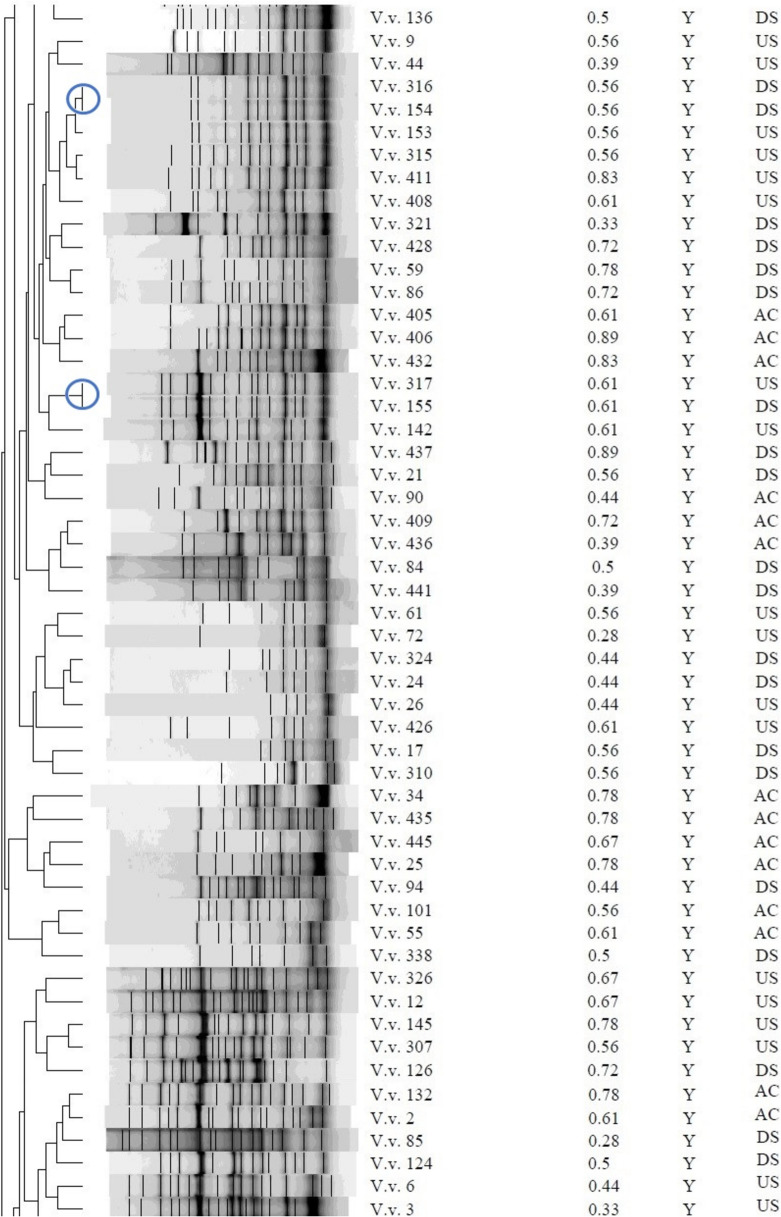

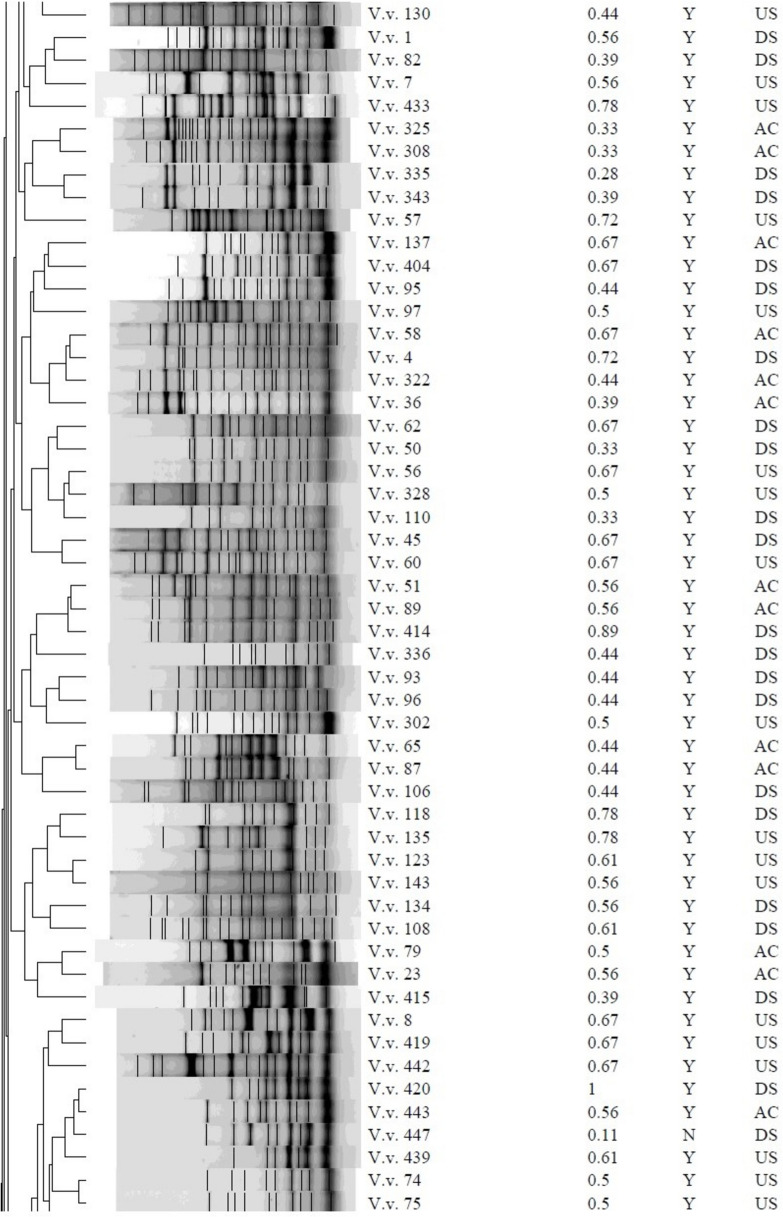

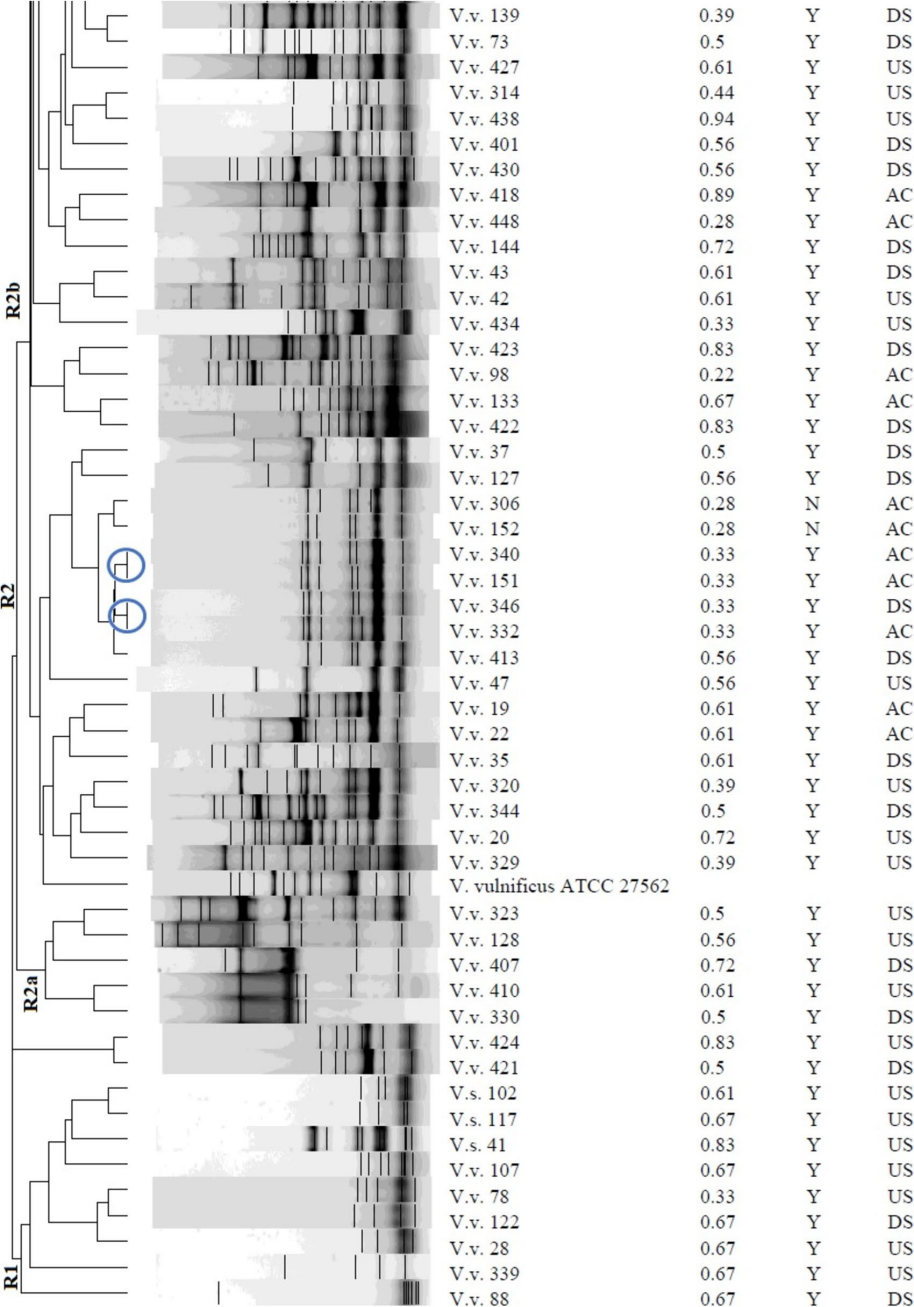
Dendrogram obtained using the Dice similarity coefficient and UPGMA analysis based on REP-PCR profiles of the *Vibrio* spp. isolated from treated effluents and upstream and downstream points of receiving rivers*.* Key: *V.v.*, *Vibrio vulnificus*; *V.a.*, *Vibrio alginolyticus; V.s.*, *Vibrio* spp., *MDR*, multi-drug-resistant-phenotype; *MARI*, multiple-antibiotic-resistance-index; *SP*, sampling point; *AC*, after chlorination; *DS*, downstream; *US*, upstream; *Y*, yes; *N*, no*.* Bacterial strains belonging to a clone are enclosed in a circle. Strains were divided into two distinct groups, *R1* and *R2*, based on a 30% similarity. The group *R*2 was further divided into *R2a* and *R2b* based on a similarity of 35%

The REP-PCR profiles and dendrogram of the *Vibrio* isolates, as shown in Fig. 5 and Figs. S3–18, demonstrated diversity within the group, with band sizes ranging from 75 to 20,000 bp. The isolates of *V. alginolyticus* exhibited a compact cluster with closely comparable genetic profiles, but the other isolates of *V. vulnificus* formed a distinct cluster, indicating substantial genetic divergence from *V. alginolyticus* After REP-PCR analysis, the strains were categorized into two separate groups: R1 and R2, based on a 30% similarity (Fig. 5). The R2 group was then subdivided into R2a and R2b due to a 35% commonality. Certain strains exhibited genetic identity with a similarity of 100%. The isolates comprised: V.a. 331, V.a. 342, V.a. 70, V.a. 140, V.v. 301, V.v. 311, V.v. 319, V.v. 71, V.v. 77, V.v. 154, V.v. 316, V.v. 155, V.v. 317, V.v. 151, V.v. 340, V.v. 332, V.v. 346, V.s. 18 and V.s. 431. Isolates that displayed similar antibiograms were categorised within the same cluster and were genetically identical. These included isolates V.a.70, V.a.140, V.v.301, V.v.319, V.v.71, V.v.77, V.v.154, V.v.316, V.v.155, V.v.317, V.v.151 and V.v.340. Nevertheless, several isolates that displayed identical fingerprints did not possess identical antibiograms, as evidenced by isolates V.a. 331 and V.a. 342, V.v. 311 differing from V.v. 301 and V.v. 319, V.v. 332 and V.v. 346, as well as V.s. 18 and V.s. 431. Isolates that displayed identical antibiograms did not necessarily possess identical genetic fingerprints, specifically isolates V.v.28, V.v.88, V.v.107, V.v.122 and V.v.339 (phenotype A38); V.v.45, V.v.58, V.v.137 and V.v.404 (phenotype A45); V.v.108, V.v.123, V.v.19, V.v.22 and V.v.35 (phenotype A50); V.v.73, V.v.74, V.v.75 and V.v.156 (phenotype B11); and V.v.65, V.v.87, V.v.93 and V.v.96 (phenotype B12).

## Discussion

The findings of this study highlight the significant influence of seasonal variations, sampling points (*SP*), and their interaction on *Vibrio* counts across the four investigated WWTPs. The interaction between season and sampling points played a significant role in influencing *Vibrio* counts across all WWTPs (Table 2) indicating that seasonal and spatial factors jointly impact the microbial dynamics within wastewater treatment systems (Beltrán de Heredia et al., 2023). Seasonal changes were shown to significantly affect *Vibrio* levels (Table 3), with the highest counts observed during spring and summer, as seen in WWTP2 and WWTP4, respectively, suggesting warmer seasons may favour the proliferation of *Vibrio* species due to increased temperatures and nutrient availability. Similarly, Osunla et al. (2021) reported *Vibrio* density in treated effluents of WWTPs was highest during the warmer summer and spring seasons.

The statistical significance of seasonal trends was evident across all WWTPs, with specific plants and sampling points showing varying levels of *Vibrio* counts. For instance, WWTP2 and WWTP4 exhibited the highest counts during spring and summer, respectively, indicating susceptibility to seasonal variations. Environmental factors such as temperature and rainfall play crucial roles in these variations. Higher temperatures during warmer seasons enhance microbial activity and nutrient availability, leading to increased *Vibrio* proliferation. Additionally, rainfall influences the dilution and transport of nutrients and microorganisms within wastewater treatment systems, further impacting *Vibrio* levels (Azuma & Hayashi, 2021; Beltrán de Heredia et al., 2023; Osunla et al., 2021). The seasonal variation of *Vibrio* species in WWTPs and aquatic environments is well-documented, with higher counts typically observed during warmer seasons (Brumfield et al., 2023; Nongogo & Okoh, 2014). Temperature plays a crucial role in *Vibrio* proliferation, with significant increases noted above 15 and 25 °C (Brumfield et al., 2023). Other environmental factors influencing *Vibrio* abundance include salinity, rainfall and nutrient availability (Chen et al., 2020). The presence of chitinous plankton, particularly copepods, is associated with higher prevalence of genes linked to clinical *Vibrio* strains (Turner et al., 2014). Different *Vibrio* species exhibit varying responses to seasonal changes, with *V*. *fluvialis*, *V*. *alginolyticus* and *V*. *splendidus* among the most sensitive to environmental fluctuations (Chen et al., 2020). The persistence of *Vibrio* pathogens in WWTP effluents suggests inadequate removal during treatment, posing potential public health risks (Nongogo & Okoh, 2014).

Sampling points also significantly influenced *Vibrio* levels across the WWTPs (Table 4). High *Vibrio* counts present in influent samples reflect the untreated nature of incoming wastewaters, whereas after chlorination samples demonstrated substantial reduction in *Vibrio* levels, highlighting the effectiveness of the disinfection process (Azuma & Hayashi, 2021). The activated sludge (*AS*) samples also showed elevated *Vibrio* counts possibly due to the concentration of organic matter and nutrients that facilitate microbial growth.

The removal efficiency of *Vibrio* spp. (Fig. 2) varied across the wastewater treatment plants (WWTPs) and seasons, highlighting differences in treatment performance and environmental influences. WWTP3 consistently demonstrated the highest removal efficiency, achieving near-complete elimination of *Vibrio* spp. across all seasons, which underscores its superior operational performance. The difference in water quality could be attributed to WWTP3 utilizing biofilters, unlike the others which employed activated sludge. Biofilters work by using biological catalysts, such as algae, bacteria, plants, protozoa and mixed microbes, to reduce unwanted pathogens such as *Vibrio* (Bai et al., 2022; Pachaiappan et al., 2022). In contrast, activated sludge systems rely on a diverse microbial community, including bacteria, fungi and protozoa (Roman et al., 2022). However, issues such as bulking sludge, caused by the overgrowth of filamentous bacteria, can significantly impair the performance of activated sludge systems resulting in poor settling and compaction (Xu et al., 2018). This ultimately affects the system’s ability to effectively remove *Vibrio* spp., contributing to the lower removal efficiencies observed in these plants.

The statistical significance of the removal efficiency data was assessed using appropriate statistical tests, confirming significant differences across the WWTPs and seasons. Specific removal efficiency values were compared with literature benchmarks, revealing that the efficiencies observed in this study are in line with global standards. For instance, WWTP3’s superior performance can be attributed to its use of biofilters, known for their high pathogen removal efficiency (Mailler et al., 2014, 2021). In contrast, the lower efficiencies observed in plants using activated sludge systems can be linked to issues such as bulking sludge, which impairs system’s performance (Ben-David et al., 2021; Kistemann et al., 2008).

Findings from this study offer significant insights into the abundance and distribution of *Vibrio* species (Fig. 3) in the examined effluents of the wastewater treatment plant and the adjacent surface waters. *V*. *vulnificus* (Fig. 3) emerged as the dominant species among the identified *Vibrio* strains, followed by *V*. *alginolyticus* (Fig. 4) and other species. In contrast, studies from Nigerian rivers identified *V*. *parahaemolyticus* as the most dominant and *V*. *vulnificus* occurring less frequently (Adesiyan et al., 2021). Similarly, research on South African final effluents reported *V*. *fluvialis* as the most frequently detected species, with lower occurrences of *V*. *vulnificus* (Conrad & Harwood, 2022; Nongogo & Okoh, 2014). The prevalence of *V. vulnificus* indicates that these waters may serve as environmental reservoirs for this pathogen*.* Notably, *V. vulnificus* are most frequently associated with human infections and provide a significant risk to human health when ingested via water or food (Mok et al., 2019; Park et al., 2018). The detection of *V. alginolyticus*, a marine pathogen, in this study indicates potential contamination of marine or estuarine environments. This discovery underscores its possible ramifications for human health and the environment (Jacobs Slifka et al., 2017). The detected abundance of *Vibrio* spp. in the tested rivers strongly suggests the potential for sporadic, unreported infections within populations or the existence of asymptomatic carriers sporadically discharging *Vibrio* into the environment as previously suggested (Nongogo & Okoh, 2014; Zheng et al., 2022). The presence of pathogenic *Vibrio* species in freshwater bodies poses risks to human health, especially with the ingestion of aquatic animals from these sources.

A significant percentage of isolates of *V. vulnificus* and *V. alginolyticus* exhibited resistance to many frequently utilized antibiotics, including ampicillin, ampicillin-sulbactam, amoxicillin clavulanate and penicillin (Table 5). All *Vibrio* spp. isolates exhibited resistance to the evaluated beta-lactam antibiotics, highlighting a uniform pattern among the species. Resistance to trimethoprim-sulfamethoxazole and sulfonamide was widespread in *V. vulnificus*, *V. alginolyticus* and *Vibrio* species. A significant percentage of isolates across all species shown resistance to imipenem, tetracycline and doxycycline. *V. vulnificus* isolates from South Korea’s coastal regions have been reported to demonstrate resistance to ampicillin, ampicillin-sulbactam, imipenem and tetracycline (Lee et al., 2021). High sensitivity to ampicillin-sulbactam and tetracycline in *V. vulnificus* isolated from water and blue crab (*Callinectes sapidus*) samples taken from the Maryland Coastal Bays has been reported (da Silva et al., 2021). The resistance rates of *Vibrio* species to tetracycline and doxycycline in this study are concerning, as these antibiotics are the primary treatments recommended for *Vibrio* infections in both adults and children, according to the Centre for Disease Control and Prevention (Yun & Kim, 2018). Consistent with previous study, this study identified several isolates exhibiting resistance to nalidixic acid (Ayodele & Okoh, 2021). Ayodele and Okoh (2021) predicted that resistance to nalidixic acid is likely to proliferate to other fluoroquinolones. Isolates exhibited significant susceptibility to amikacin and gentamicin, consistent with previous findings (Adesiyan et al., 2021), where elevated susceptibility to these antibiotics has been reported. Ciprofloxacin and ofloxacin showed comparable efficacy, with 58.4% and 65.5% susceptibility, respectively, observed for *V. vulnificus* isolates. This contrasts with another study that reported lower susceptibility in *V*. *vulnificus* (*n* = 37) isolated from a watershed in a rustic milieu, showing 64.9% for ciprofloxacin and 29.7% for ofloxacin (Gxalo et al., 2021).

The resistance to beta-lactams observed in this study may correlate with antibiotic pollution in wastewater, as these antibiotics are commonly used and can enter aquatic environments through various pathways (Kumarage et al., 2022; Loo et al., 2020). Environmental sources contributing to antibiotic resistance include agricultural runoff, industrial discharges and improper disposal of pharmaceuticals. The report on imipenem resistance is clinically significant, as imipenem is a last-resort antibiotic for treating severe bacterial infections. Comparing resistance patterns with global data reveals that the rise of antibiotic resistance in the *Vibrio* population is a growing public health concern (Kumarage et al., 2022). Consequently, the prudent application of antibiotics and ongoing monitoring is essential to mitigate the development of multi-drug-resistant *Vibrio* infections that pose a threat to public health. These findings underscore the urgency of addressing antibiotic resistance in the *Vibrio* population to ensure effective treatment options remain available (Kumarage et al., 2022; Loo et al., 2020).

Furthermore, 155 antibiotic resistance patterns were identified across the investigated *Vibrio* species, with 98% (*n* = 200) of the *Vibrio* isolates exhibiting resistance to three or more antibiotics (Table S1). This signifies that the isolates exhibit resistance to antibiotics that are clinically significant for therapy. A *MARI* index of 0.2 indicates a setting with prevalent antibiotic usage (Olonitola et al., 2015). Among the 200 *Vibrio* strains recovered in this investigation, 199 exhibited a *MAR* score of ≥ 0.2, categorising them as high risk. Significant multi-drug resistance has been documented in both environmental and clinical *V. vulnificus* and other *Vibrio* species (Baker-Austin et al., 2009; Shaw et al., 2014; Yang et al., 2017). Conversely, recent research indicated that multi-drug resistance was present in merely 38% (*n* = 50) of *V. alginolyticus* isolated from freshwater and marine fish and shellfish (Sadat et al., 2021). These data show improper antibiotic usage in the environment. An elevated *MARI* value, as noted in this study, may stem from anthropogenic activities in the environment, signifying significant contamination with antimicrobial compounds (Ayodele & Okoh, 2021).

The REP-PCR study enabled the distinction of closely related *Vibrio* species, offering insights into the genetic diversity among *V. alginolyticus* and *V. vulnificus* isolates (Fig. 5). The identified similarities and differences between genetic fingerprints and antibiograms emphasise the intricacy of microbial diversity and the necessity of utilising various analytical methods for a whole comprehension of bacterial communities. The genetically identical strains identified, including V.a. 331 (*US*) and V.a. 342 (*DS*), V.v. 301 (*DS*), V.v. 311 (*AC*) and V.v. 319 (*DS*), V.v. 71 (*AC*) and V.v. 77 (*DS*), V.v. 155 (*DS*) and V.v. 317 (*US*), V.v. 332 (*AC*) and V.v. 346 (*DS*), as well as V.s. 18 (*DS*) and V.s. 431 (*US*), were isolated from various sampling locations, indicating a shared origin of the isolates. Strains identified in the treated effluent and *DS* indicate that the treated effluent may have contributed to the increased number of *Vibrio* strains in the receiving rivers, underscoring a possible risk to human and environmental health (Osunla et al., 2021). The occurrence of a strain in both upstream and downstream sites may result from natural dispersal, wherein the microbe is transported by water flow from upstream to downstream (Wang et al., 2021).

Clonal associations categorised by isolates within the same cluster also displaying identical antibiograms suggest they have the same source of origin (Havenga et al., 2022). However, horizontal gene transfer can introduce variations such as antibiotic resistance genes, leading to varied antibiograms among isolates with identical genetic fingerprints (Abioye et al., 2021). This mechanism may result in variations in the antibiogram while preserving overall genetic similarity. Furthermore, these isolates may respond differentially to selective pressures or environmental stimuli, leading to differences in the manifestation of antibiotic resistance. Environmental factors, including competing microbes and fluctuating nutrition levels, can influence the expression of antibiotic resistance genes (Fletcher, 2015). Conversely, exhibition of identical antibiograms in isolates with different genetic profiles may be due to the acquisition of analogous antibiotic resistance genes by horizontal gene transfer, resulting in common behavioural characteristics (Tao et al., 2022).

## Conclusion

This study revealed a consistent reduction in the concentration of the *Vibrio* population in treated effluent compared to influent samples across four wastewater treatment plants in Durban, KwaZulu-Natal. However, the dominance of *Vibrio vulnificus* and *Vibrio alginolyticus* in the treated effluents and receiving rivers suggest the potential role of these waters as environmental reservoirs, constituting a significant public health risk to individuals who use or consume water from these sources given the pathogenic nature of these species. The study also identified a concerning trend of multi-drug resistance among *Vibrio* species, highlighting the importance of holistic approaches to develop effective treatment strategies. REP-PCR provided valuable insights into the genetic diversity of *V. alginolyticus* and *V. vulnificus* isolates. Notably, the identification of genetically identical strains from different sampling points, particularly between treated effluents and downstream locations, suggests that treated effluent serves as a potential source for the spread of *Vibrio* strains in receiving rivers. In addition to *V. alginolyticus* and *V. vulnificus*, other *Vibrio* species were isolated from the samples. Future studies may include identifying these isolates, through PCR amplification using species-specific primers, aiding the understanding of microbial diversity in treated wastewater effluents and environmental waters. This may help uncover potential health implications associated with their presence, including the risk of waterborne diseases. Another key focus should be identifying antibiotic resistance genes and the underlying mechanisms responsible for the multi-drug resistance observed within the isolates. Additionally, whole-genome sequencing will be essential to provide an in-depth understanding of the metabolic and functional capabilities of these isolates. To mitigate the public health risks associated with *Vibrio* species in treated effluents, policy recommendations should include implementation of advanced wastewater treatment technologies, regular monitoring of effluent quality and the development of guidelines for safe discharge of treated effluents into receiving water bodies. These measures will help ensure the effectiveness of wastewater treatment processes and protect public health.

## Supplementary Information

Below is the link to the electronic supplementary material.

Supplementary file1 (PDF 1.47 MB)

## Data Availability

No datasets were generated or analysed during the current study.
