# Supplementary material for: Seasonal variability, antibiogram and genetic diversity of Vibrio spp. recovered from effluent discharge of wastewater treatment plants and their receiving rivers in Durban, South Africa
Source: Environ Monit Assess. 2025 Jun 10;197(7):745. doi: 10.1007/s10661-025-14171-7 (PMC12152032; doi:10.1007/s10661-025-14171-7)
Supplement: Supplementary file 1 — Supplementary file1 (PDF 1.47 MB) [file 10661_2025_14171_MOESM1_ESM.pdf]

SUPPLEMENTARY MATERIAL

Journal: Environmental Monitoring and Assessment

Seasonal Variability, Antibiogram and Genetic Diversity of *Vibrio* spp. Recovered from Effluent Discharge of Wastewater Treatment Plants and their Receiving Rivers in Durban, South Africa

Kerisha Ramessar, Ademola O. Olaniran*

Discipline of Microbiology, School of Life Sciences, University of KwaZulu-Natal (Westville Campus), Durban, 4000, South Africa

*Corresponding author:

Email: olanirana@ukzn.ac.za, Tel.: +27-31-260-7400.

Table S1: Distribution of antibiotic resistance phenotype, MARI, virulence genes and MVGI amongst *V. vulnificus* isolates (n=178)

Isolate no:	Phenotype	Resistance profile	MARI
V.v.420	A1	AMP10 SAM20 P10 AK30 CN10 FOX30 CTX30 CAZ30 C30 NA30 CIP5 DO30 OFX5 TE30 SXT25 AMC30 IPM10 S300	1
V.v.438	A2	AMP10 SAM20 P10 AK30 CN10 FOX30 CTX30 CAZ30 C30 NA30 CIP5 DO30 OFX5 TE30 AMC30 IPM10 S300	0,94
V.v.20	A3	AMP10 SAM20 P10 AK30 CN10 FOX30 CTX30 CAZ30 DO30 TE30 AMC30 IPM10 S300	0,72
V.v.57	A4	AMP10 SAM20 P10 AK30 CN10 FOX30 C30 DO30 TE30 SXT25 AMC30 IPM10 S300	0,72
V.v.418	A5	AMP10 SAM20 P10 AK30 CN10 CTX30 CAZ30 C30 CIP5 DO30 OFX5 TE30 SXT25 AMC30 IPM10 S300	0,89
V.v.32	A6	AMP10 SAM20 P10 AK30 CN10 CTX30 CAZ30 NA30 SXT25 AMC30 IPM10	0,61
V.v.417	A7	AMP10 SAM20 P10 AK30 FOX30 CTX30 CAZ30 C30 NA30 CIP5 DO30 OFX5 TE30 SXT25 AMC30 IPM10 S300	0,94
V.v.406	A8	AMP10 SAM20 P10 AK30 FOX30 CTX30 CAZ30 C30 NA30 CIP5 DO30 OFX5 TE30 SXT25 AMC30 IPM10	0,89
V.v.25	A9	AMP10 SAM20 P10 AK30 FOX30 CTX30 CAZ30 C30 DO30 TE30 SXT25 AMC30 IPM10 S300	0,78
V.v.411	A10	AMP10 SAM20 P10 AK30 FOX30 CTX30 CAZ30 NA30 CIP5 DO30 OFX5 TE30 AMC30 IPM10 S300	0,83
V.v.405	A11	AMP10 SAM20 P10 AK30 FOX30 CTX30 CAZ30 CIP5 AMC30 IPM10 S300	0,61
V.v.101	A12	AMP10 SAM20 P10 AK30 FOX30 CTX30 CAZ30 AMC30 IPM10 S300	0,56
V.v.118<br>V.v.135	A13	AMP10 SAM20 P10 AK30 FOX30 CTX30 CAZ30 NA30 DO30 TE30 SXT25 AMC30 IPM10 S300	0,78
V.v.424	A14	AMP10 SAM20 P10 AK30 FOX30 CTX30 NA30 CIP5 DO30 OFX5 TE30 SXT25 AMC30 IPM10 S300	0,83
V.v.126	A15	AMP10 SAM20 P10 AK30 FOX30 CTX30 NA30 DO30 TE30 SXT25 AMC30 IPM10 S300	0,72
V.v.133	A16	AMP10 SAM20 P10 AK30 FOX30 CTX30 DO30 TE30 SXT25 AMC30 IPM10 S300	0,67
V.v.55	A17	AMP10 SAM20 P10 AK30 FOX30 CTX30 DO30 TE30 SXT25 AMC30 S300	0,61
V.v.134	A18	AMP10 SAM20 P10 AK30 FOX30 CTX30 OFX5 AMC30 IPM10 S300	0,56
V.v.43<br>V.v.42	A19	AMP10 SAM20 P10 AK30 FOX30 DO30 TE30 SXT25 AMC30 IPM10 S300	0,61
V.v.346	A20	AMP10 SAM20 P10 AK30 FOX30 AMC30	0,33
V.v.439	A21	AMP10 SAM20 P10 AK30 CTX30 CAZ30 C30 DO30 TE30 AMC30 IPM10	0,61

V.v.128	A22	AMP10 SAM20 P10 AK30 CTX30 DO30 TE30 AMC30 IPM10 S300	0,56
V.v.437	A23	AMP10 SAM20 P10 CN10 FOX30 CTX30 CAZ30 NA30 CIP5 DO30 OFX5 TE30 SXT25 AMC30 IPM10 S300	0,89
V.v.341	A24	AMP10 SAM20 P10 CN10 FOX30 CTX30 CAZ30 DO30 TE30 SXT25 AMC30 S300	0,67
V.v.60	A25	AMP10 SAM20 P10 CN10 FOX30 CTX30 C30 DO30 TE30 AMC30 IPM10 S300	0,67
V.v.52	A26	AMP10 SAM20 P10 CN10 FOX30 CTX30 NA30 DO30 TE30 AMC30 IPM10 S300	0.67
V.v.2	A27	AMP10 SAM20 P10 CN10 FOX30 C30 DO30 TE30 SXT25 AMC30 S300	0.61
V.v.56<br>V.v.62	A28	AMP10 SAM20 P10 CN10 FOX30 C30 DO30 TE30 SXT25 AMC30 IPM10 S300	0,67
V.v.61	A29	AMP10 SAM20 P10 CN10 C30 DO30 TE30 SXT25 AMC30 S300	0.56
V.v.414	A30	AMP10 SAM20 P10 FOX30 CTX30 CAZ30 C30 NA30 CIP5 DO30 OFX5 TE30 SXT25 AMC30 IPM10 S300	0.89
V.v.432	A31	AMP10 SAM20 P10 FOX30 CTX30 CAZ30 NA30 CIP5 DO30 OFX5 TE30 SXT25 AMC30 IPM10 S300	0,83
V.v.145	A32	AMP10 SAM20 P10 FOX30 CTX30 CAZ30 NA30 CIP5 DO30 TE30 SXT25 AMC30 IPM10 S300	0,78
V.v.445	A33	AMP10 SAM20 P10 FOX30 CTX30 CAZ30 NA30 CIP5 TE30 AMC30 IPM10 S300	0,67
V.v.428	A34	AMP10 SAM20 P10 FOX30 CTX30 CAZ30 NA30 DO30 TE30 SXT25 AMC30 IPM10 S300	0,72
V.v.348	A35	AMP10 SAM20 P10 FOX30 CTX30 CAZ30 NA30 DO30 TE30 SXT25 AMC30 S300	0,67
V.v.59	A36	AMP10 SAM20 P10 FOX30 CTX30 CAZ30 NA30 DO30 OFX5 TE30 SXT25 AMC30 IPM10 S300	0,78
V.v.132	A37	AMP10 SAM20 P10 FOX30 CTX30 CAZ30 CIP5 DO30 OFX5 TE30 SXT25 AMC30 IPM10 S300	0,78
V.v.28<br>V.v.88<br>V.v.107<br>V.v.122<br>V.v.339	A38	AMP10 SAM20 P10 FOX30 CTX30 CAZ30 DO30 TE30 SXT25 AMC30 IPM10 S300	0,67
V.v.410	A39	AMP10 SAM20 P10 FOX30 CTX30 CAZ30 DO30 TE30 AMC30 IPM10 S300	0,61
V.v.21	A40	AMP10 SAM20 P10 FOX30 CTX30 CAZ30 TE30 SXT25 AMC30 S300	0,56
V.v.37	A41	AMP10 SAM20 P10 FOX30 CTX30 CAZ30 AMC30 IPM10 S300	0,50
V.v.422<br>V.v.423	A42	AMP10 SAM20 P10 FOX30 CTX30 C30 NA30 CIP5 DO30 OFX5 TE30 SXT25 AMC30 IPM10 S300	0,83
V.v.435<br>V.v.34	A43	AMP10 SAM20 P10 FOX30 CTX30 C30 NA30 CIP5 DO30 TE30 SXT25 AMC30 IPM10 S300	0,78
V.v.120	A44	AMP10 SAM20 P10 FOX30 CTX30 C30 NA30 DO30 TE30 SXT25 AMC30 IPM10 S300	0,72

V.v.129<br>V.v.131<br>V.v.144<br>V.v.45<br>V.v.58<br>V.v.137<br>V.v.404	A45	AMP10 SAM20 P10 FOX30 CTX30 C30 DO30 TE30 SXT25 AMC30 IPM10 S300	0.67
V.v.433	A46	AMP10 SAM20 P10 FOX30 CTX30 NA30 CIP5 DO30 OFX5 TE30 SXT25 AMC30 IPM10 S300	0,78
V.v.86	A47	AMP10 SAM20 P10 FOX30 CTX30 NA30 CIP5 DO30 TE30 SXT25 AMC30 IPM10 S300	0,72
V.v.12<br>V.v.326	A48	AMP10 SAM20 P10 FOX30 CTX30 NA30 DO30 TE30 SXT25 AMC30 IPM10 S300	0,67
V.v.139	A49	AMP10 SAM20 P10 FOX30 CTX30 NA30 SXT25	0,39
V.v.108<br>V.v.123<br>V.v.19<br>V.v.22<br>V.v.35	A50	AMP10 SAM20 P10 FOX30 CTX30 DO30 TE30 SXT25 AMC30 IPM10 S300	0,61
V.v.17<br>V.v.310	A52	AMP10 SAM20 P10 FOX30 CTX30 DO30 TE30 SXT25 AMC30 S300	0,56
V.v.9	A53	AMP10 SAM20 P10 FOX30 CTX30 DO30 TE30 SXT25 IPM10 S300	0,56
V.v.338	A54	AMP10 SAM20 P10 FOX30 CTX30 DO30 TE30 AMC30 S300	0,50
V.v.79	A55	AMP10 SAM20 P10 FOX30 CTX30 DO30 AMC30 IPM10 S300	0,50
V.v.324	A56	AMP10 SAM20 P10 FOX30 CTX30 AMC30 IPM10 S300	0,44
V.v.311	A57	AMP10 SAM20 P10 FOX30 CTX30 AMC30 S300	0,39
V.v.302	A58	AMP10 SAM20 P10 FOX30 CTX30 CAZ30 AMC30 IPM10 S300	0,50
V.v.8<br>V.v.419<br>V.v.442	A59	AMP10 SAM20 P10 FOX30 C30 NA30 DO30 TE30 SXT25 AMC30 IPM10 S300	0,67
V.v.444	A60	AMP10 SAM20 P10 FOX30 C30 NA30 DO30 TE30 SXT25 AMC30	0,56
V.v.427	A61	AMP10 SAM20 P10 FOX30 C30 DO30 TE30 SXT25 AMC30 IPM10 S300	0,61
V.v.328	A62	AMP10 SAM20 P10 FOX30 C30 DO30 TE30 AMC30 S300	0,50
V.v.24	A63	AMP10 SAM20 P10 FOX30 C30 AMC30 IPM10 S300	0,44

V.v.26<br>V.v.318<br>V.v.153	A64	AMP10 SAM20 P10 FOX30 NA30 DO30 TE30 SXT25 AMC30 IPM10 S300	0,61
V.v.154<br>V.v.315	A65	AMP10 SAM20 P10 FOX30 NA30 DO30 TE30 SXT25 AMC30 S300	0,56
V.v.316<br>V.v.413	A66	AMP10 SAM20 P10 FOX30 NA30 TE30 SXT25 AMC30 IPM10 S300	0,56
V.v.314	A67	AMP10 SAM20 P10 FOX30 NA30 AMC30 IPM10 S300	0,44
V.v.303	A68	AMP10 SAM20 P10 FOX30 NA30 AMC30 S300	0,39
V.v.332	A69	AMP10 SAM20 P10 FOX30 NA30 AMC30	0,33
V.v.1<br>V.v.7	A70	AMP10 SAM20 P10 FOX30 DO30 TE30 SXT25 AMC30 IPM10 S300	0,56
V.v.323<br>V.v.330	A71	AMP10 SAM20 P10 FOX30 DO30 TE30 SXT25 AMC30 S300	0.50
V.v.319	A72	AMP10 SAM20 P10 FOX30 DO30 TE30 AMC30 IPM10 S300	0,50
V.v.301	A73	AMP10 SAM20 P10 FOX30 DO30 TE30 AMC30 S300	0,44
V.v.138	A74	AMP10 SAM20 P10 FOX30 DO30 AMC30 IPM10 S300	0,44
V.v.308<br>V.v.325	A75	AMP10 SAM20 P10 FOX30 AMC30 S300	0.33
V.v.327	A76	AMP10 SAM20 P10 FOX30 AMC30	0,28
V.v.47	A77	AMP10 SAM20 P10 FOX30 DO30 TE30 SXT25 AMC30 IPM10 S300	0,56
V.v.304<br>V.v.336	A78	AMP10 SAM20 P10 FOX30 DO30 TE30 AMC30 S300	0,44
V.v.322	A79	AMP10 SAM20 P10 C30 DO30 TE30 AMC30 S300	0,44
V.v.320<br>V.v.329	A80	AMP10 SAM20 P10 DO30 TE30 AMC30 S300	0,39
V.v.90	A81	AMP10 SAM20 P10 FOX30 SXT25 AMC30 IPM10 S300	0,44
V.v.151<br>V.v.340	A82	AMP10 SAM20 P10 FOX30 AMC30 S300	0,33
V.v.152<br>V.v.306	A83	AMP10 SAM20 P10 FOX30 AMC30	0,28

V.v.81	A84	AMP10 SAM20 P10 FOX30 SXT25 AMC30 S300	0,39
V.v.48	A85	AMP10 SAM20 P10 FOX30 AMC30 IPM10 S300	0,39
V.v.409	A86	AMP10 SAM20 P10 CTX30 CAZ30 NA30 CIP5 DO30 OFX5 TE30 AMC30 IPM10 S300	0,72
V.v.407	A87	AMP10 SAM20 P10 CTX30 CAZ30 NA30 CIP5 DO30 OFX5 TE30 SXT25 AMC30 IPM10	0,72
V.v.408	A88	AMP10 SAM20 P10 CTX30 CAZ30 CIP5 DO30 TE30 AMC30 IPM10 S300	0,61
V.v.426	A89	AMP10 SAM20 P10 CTX30 NA30 CIP5 OFX5 SXT25 AMC30 IPM10 S300	0,61
V.v.443	A90	AMP10 SAM20 P10 CTX30 NA30 CIP5 OFX5 AMC30 IPM10 S300	0,56
V.v.415	A91	AMP10 SAM20 P10 CTX30 NA30 AMC30 IPM10	0,39
V.v.136	A92	AMP10 SAM20 P10 CTX30 DO30 TE30 AMC30 IPM10 S300	0,5
V.v.29	A93	AMP10 SAM20 P10 CTX30 DO30 TE30 AMC30 S300	0,44
V.v.401	A94	AMP10 SAM20 P10 NA30 CIP5 OFX5 TE30 AMC30 IPM10 S300	0,56
V.v.421	A95	AMP10 SAM20 P10 NA30 TE30 SXT25 AMC30 IPM10 S300	0,50
V.v.430	A96	AMP10 SAM20 P10 NA30 DO30 OFX5 TE30 AMC30 IPM10 S300	0,56
V.v.51<br>V.v.89	A97	AMP10 SAM20 P10 FOX30 DO30 TE30 SXT25 AMC30 IPM10 S300	0,56
V.v.434	A98	AMP10 SAM20 P10 DO30 AMC30 IPM10	0,33
V.v.436<br>V.v.441	A99	AMP10 SAM20 P10 NA30 AMC30 IPM10 S300	0,39
V.v.143	B1	AMP10 P10 AK30 FOX30 CTX30 CAZ30 NA30 AMC30 IPM10 S300	0,56
V.v.106	B2	AMP10 P10 AK30 FOX30 CTX30 CAZ30 IPM10 S300	0,44
V.v.4	B3	AMP10 P10 CN10 FOX30 CTX30 CAZ30 C30 DO30 TE30 SXT25 AMC30 IPM10 S300	0,72
V.v.412	B4	AMP10 P10 FOX30 CTX30 CAZ30 C30 CIP5 DO30 TE30 SXT25 AMC30 IPM10 S300	0,72
V.v.71<br>V.v.77<br>V.v.157	B5	AMP10 P10 FOX30 CTX30 CAZ30 DO30 TE30 SXT25 AMC30 IPM10 S300	0,61
V.v.127	B6	AMP10 P10 FOX30 NA30 DO30 TE30 SXT25 AMC30 IPM10 S300	0,56
V.v.142<br>V.v.155<br>V.v.317	B7	AMP10 P10 FOX30 CTX30 NA30 DO30 TE30 SXT25 AMC30 IPM10 S300	0,61
V.v.23	B8	AMP10 P10 FOX30 CTX30 DO30 TE30 SXT25 AMC30 IPM10 S300	0,56
V.v.344	B9	AMP10 P10 CN10 FOX30 DO30 TE30 SXT25 AMC30 S300	0,50

V.v.307	B10	AMP10 P10 FOX30 NA30 DO30 TE30 SXT25 AMC30 IPM10 S300	0,56
V.v.73<br>V.v.74<br>V.v.75<br>V.v.156	B11	AMP10 P10 FOX30 DO30 TE30 SXT25 AMC30 IPM10 S300	0,5
V.v.65<br>V.v.87<br>V.v.93<br>V.v.96	B12	AMP10 P10 FOX30 DO30 TE30 SXT25 AMC30 S300	0,44
V.v.94	B13	AMP10 P10 FOX30 DO30 TE30 SXT25 IPM10 S300	0,44
V.v.82	B14	AMP10 P10 FOX30 SXT25 AMC30 IPM10 S300	0,38
V.v.98	B15	AMP10 P10 FOX30 S300	0,22
V.v.85	B16	AMP10 P10 DO30 TE30 S300	0,28
V.v.78	B17	AMP10 P10 FOX30 AMC30 IPM10 S300	0,33
V.v.72	B18	AMP10 P10 FOX30 AMC30 S300	0,28
V.v.335	B19	AMP10 P10 FOX30 NA30 S300	0,28
V.v.97	B20	AMP10 P10 CTX30 C30 DO30 TE30 SXT25 IPM10 S300	0,5
V.v.84	B21	AMP10 P10 CTX30 NA30 DO30 TE30 SXT25 IPM10 S300	0,5
V.v.6<br>V.v.130	B22	AMP10 P10 NA30 DO30 TE30 SXT25 AMC30 S300	0,44
V.v.321	B23	AMP10 P10 NA30 DO30 TE30 AMC30	0,33
V.v.44	B24	SAM20 P10 C30 DO30 TE30 AMC30 S300	0,39
V.v.50	B25	SAM20 P10 C30 DO30 TE30 AMC30	0,33
V.v.343	C1	P10 AK30 FOX30 CAZ30 NA30 AMC30 S300	0,39
V.v.36	C2	P10 CN10 FOX30 C30 DO30 TE30 S300	0,39
V.v.3	C3	P10 CN10 C30 DO30 TE30 S300	0,33
V.v.124	C4	P10 FOX30 DO30 OFX5 TE30 SXT25 AMC30 IPM10 S300	0,50
V.v.95	C5	P10 FOX30 DO30 TE30 SXT25 AMC30 IPM10 S300	0,44
V.v.448	C6	P10 CTX30 DO30 IPM10 S300	0,28
V.v.110	C7	P10 DO30 TE30 AMC30 IPM10 S300	0,33
V.v.447	C8	P10 S300	0,11

V.a.111	D1	AMP10 SAM20 P10 AK30 FOX30 CTX30 CAZ30 NA30 CIP5 SXT25 AMC30 IPM10 S300	0,72
V.a.416	D2	AMP10 SAM20 P10 AK30 FOX30 CTX30 CAZ30 NA30 CIP5 DO30 OFX5 TE30 SXT25 AMC30 IPM10 S300	0,89
V.a.429	D3	AMP10 SAM20 P10 AK30 CN10 FOX30 CTX30 CAZ30 NA30 CIP5 OFX5 SXT25 S300	0,72
V.a.342	D4	AMP10 SAM20 P10 CN10 FOX30 CTX30 NA30 DO30 TE30 SXT25 AMC30 IPM10 S300	0,72
V.a.425	A30	AMP10 SAM20 P10 FOX30 CTX30 CAZ30 C30 NA30 CIP5 DO30 OFX5 TE30 SXT25 AMC30 IPM10 S300	0.89
V.a.64	D6	AMP10 SAM20 P10 FOX30 CTX30 CIP5 DO30 TE30 SXT25 AMC30 S300	0,61
V.a.40	A64	AMP10 SAM20 P10 FOX30 NA30 DO30 TE30 SXT25 AMC30 IPM10 S300	0,61
V.a.331	E1	AMP10 P10 AK30 FOX30 NA30 AMC30 S300	0,39
V.a.80	E2	AMP10 P10 FOX30 CTX30 AMC30 IPM10 S300	0,39
V.a.33<br>V.a.70<br>V.a.83<br>V.a.140	B8	AMP10 P10 FOX30 CTX30 DO30 TE30 SXT25 AMC30 IPM10 S300	0,56
V.a.67	E4	AMP10 P10 FOX30 DO30 AMC30 IPM10 S300	0,39
V.a.53	E5	AMP10 P10 CTX30 S300	0,22
V.s.15	A39	AMP10 SAM20 P10 FOX30 CTX30 CAZ30 DO30 TE30 AMC30 IPM10 S300	0,61
V.s.18	F2	AMP10 SAM20 P10 CTX30 CAZ30 DO30 TE30 AMC30 IPM10 S300	0,56
V.s.41	F3	AMP10 SAM20 P10 AK30 CN10 FOX30 CTX30 CAZ30 CIP5 DO30 TE30 SXT25 AMC30 IPM10 S300	0,83
V.s.117	A38	AMP10 SAM20 P10 FOX30 CTX30 CAZ30 DO30 TE30 SXT25 AMC30 IPM10 S300	0,67
V.s.102	A50	AMP10 SAM20 P10 FOX30 CTX30 DO30 TE30 SXT25 AMC30 IPM10 S300	0,61
V.s.49	A64	AMP10 SAM20 P10 FOX30 NA30 DO30 TE30 SXT25 AMC30 IPM10 S300	0,61
V.s.431	F7	AMP10 SAM20 P10 CTX30 NA30 CIP5 OFX5 TE30 SXT25 AMC30 IPM10	0,61

Key: V.v.- *Vibrio vulnificus*, V.a- *Vibrio alginolyticus*, V.s.- *Vibrio* spp., AMP10-ampicllin, SAM20- ampicillin sulbactam, P10- penicillin, AK30- amikacin, CN10- gentamicin, FOX30- cefoxitin, CTX30- cefotaxime, CAZ30- ceftazimide, C30- chloramphenicol, NA30- nalidixic acid, CIP5- ciprofloxacin, DO30- deoxycycline, OFX5- oflaxacin, TE30- tetracycline, SXT25- trimethoprim-sulfamethoxazole, AMC30- amoxicillin-clavulanate, IPM10-imipenem, S300- sulfonamide, MDRP- multi-drug-resistant-phenotype, MARI- multiple-antibiotic-resistance-inde

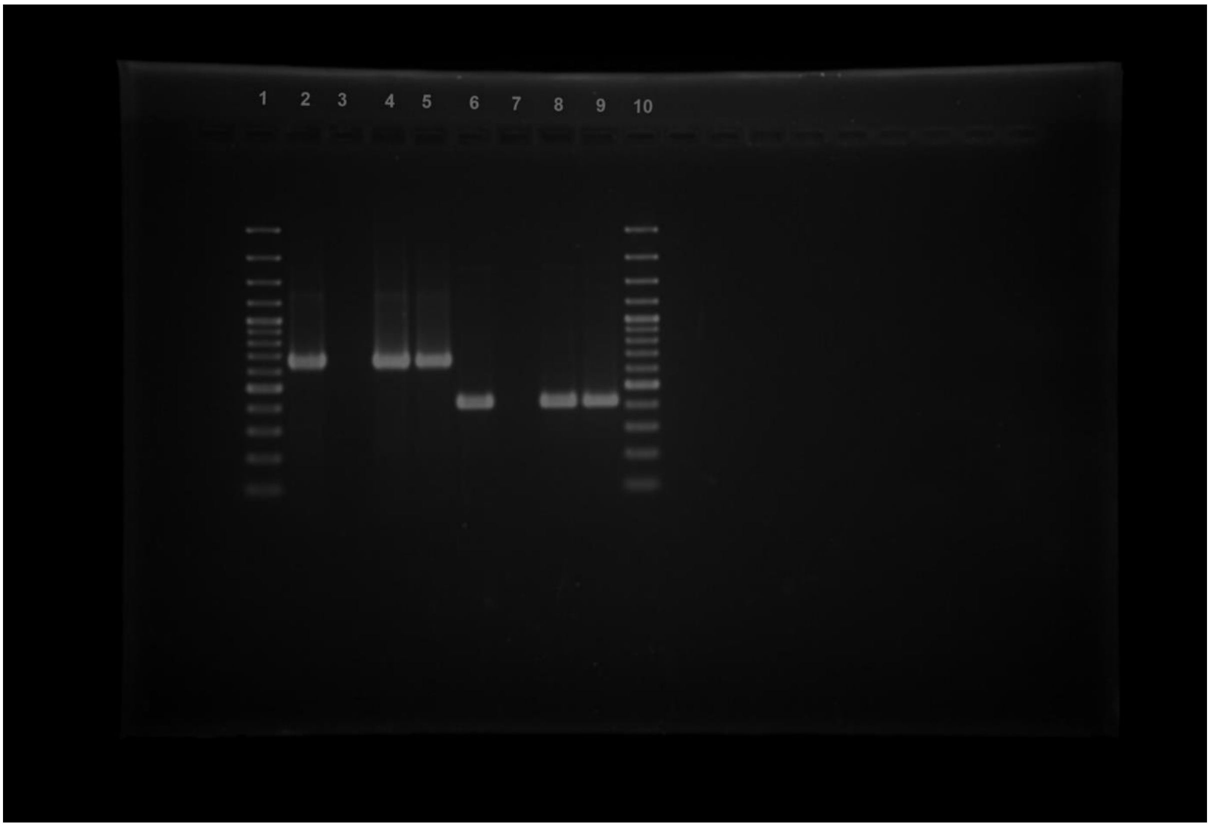

Fig. S1: Representative gel of amplified *V16S* gene (663bp) for *Vibrio* genus identification and *Vvhsp* (410bp) for identification of *Vibrio vulnificus* (V.v.) in isolates. Lanes 1 and 10 show the 100bp molecular marker. Lane 2 shows the PCR product for *V16s* gene (663bp) in the positive control (*V. vulnificus* ATCC 27562), lane 3 shows the negative control and lanes 4 and 5 shows the PCR product for *V16S* gene (663 bp) in the representative isolates V.v.419 and V.v.420. Lane 6 shows the PCR product for the *Vvhsp* gene (410 bp) in the positive control (*V. vulnificus* ATCC 27562), lane 7 shows the negative control and lanes 8 and 9 shows the PCR product for *Vvhsp* gene (410bp)in the representative isolates V.v.419 and V.v.420.

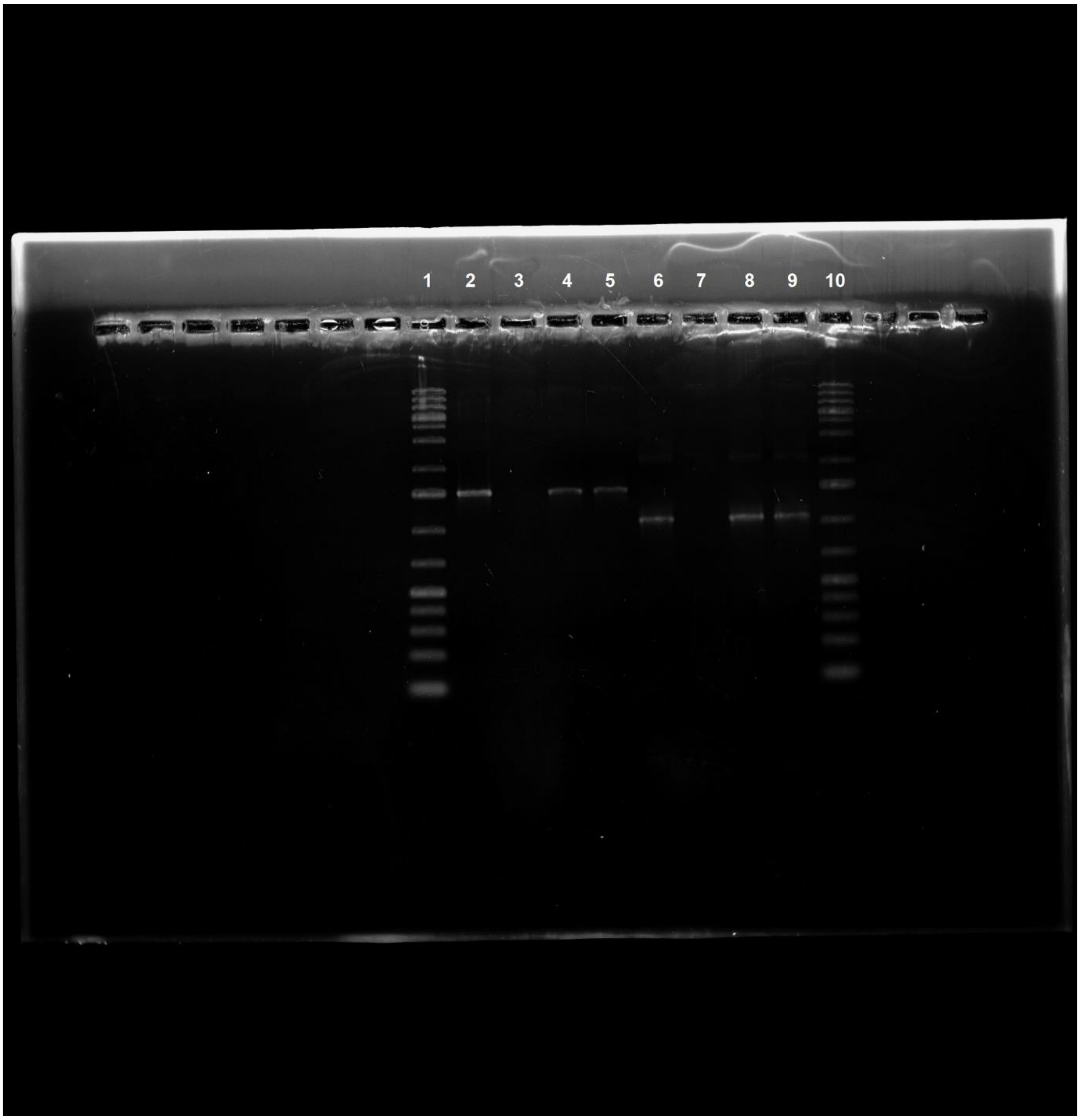

Fig. S2: Representative gel showing semi-nested PCR products for *VA16F1* (1500 bp) and *VA16F2* (1000bp) for identification *of Vibrio alginolyticus* (V.a.). Lanes 1 and 10 show the 1kb molecular marker. Lane 2 shows the PCR product for *VA16F1* (1500bp) for the positive control (*V. alginolyticus* ATCC 17749), lane 3 shows the negative control and lanes 4 and 5 shows the PCR product for *VA16F1* (1500bp) in the representative isolates V.a.111 and V.a.416. Lane 6 shows the PCR product for *VA16F2* (1000bp) for the positive control, lane 7 shows the negative control and lanes 8 and 9 shows the PCR product for *VA16F2* (1000bp) in the representative isolates V.a.111 and V.a.416.

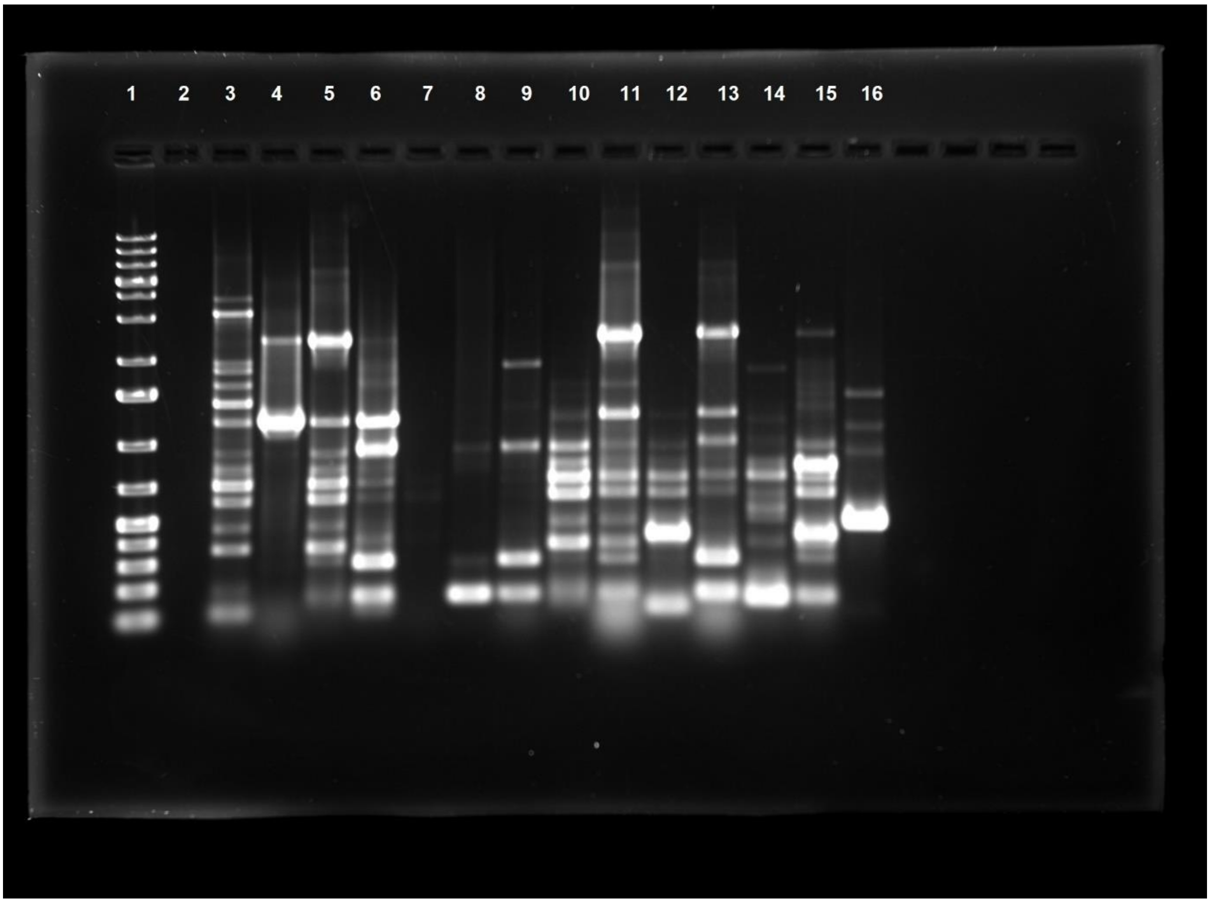

Fig. S3: REP-PCR profiles of the *Vibrio spp*. isolated from treated effluents and upstream and downstream points of receiving rivers. The lanes represent: 1kb molecular weight marker (lane 1), negative control (lane 2), V.v.433(lane 3), V.v.411 (lane 4), V.v.414 (lane 5), V.v. 424 (lane 6), V.v.413(lane 7), V.v.422 (lane 8), V.v.404 (lane 9), V.v.420 (lane 10), V.v. 418 (lane 11), V.v.434 (lane 12), V.v.412 (lane 13), V.v.445 (lane 14), V.v.415(lane 15) and positive control (lane 16).

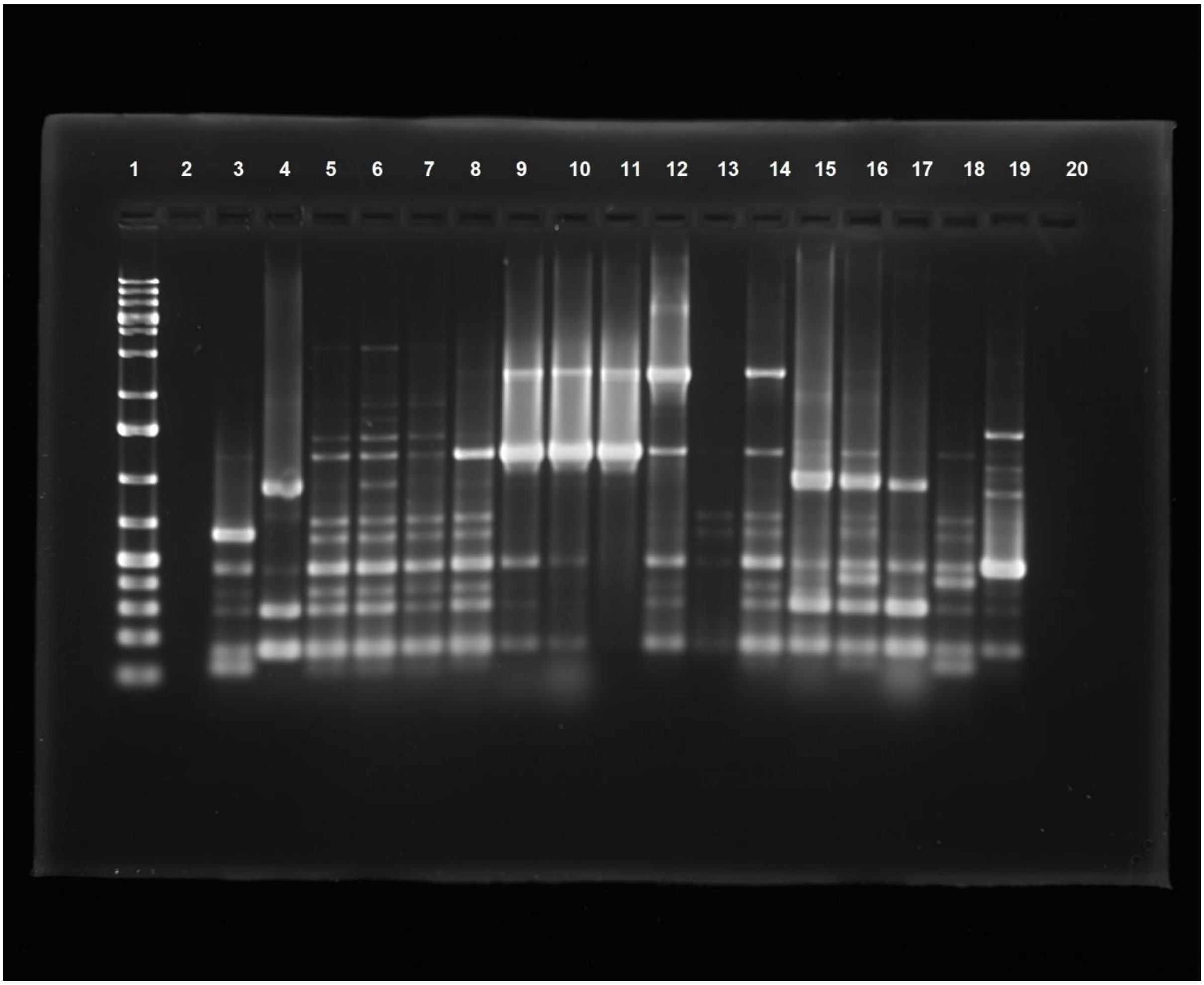

Fig. S4: REP-PCR profiles of the *Vibrio spp*. isolated from treated effluents and upstream and downstream points of receiving rivers. The lanes represent: 1 kb molecular weight marker (lane 1), negative control (lane 2), V.v.401 (lane 3), V.v.404 (lane 4), V.v.405 (lane 5), V.v.406 (lane 6), V.v.408 (lane 7), V.v.409 (lane 8), V.v.410 (lane 9), V.v.411 (lane 10), V.v.412 (lane 11), V.v.415 (lane 12), V.v.417 (lane 13), V.v.419 (lane 14), V.v.421 (lane 15), V.v.422 (lane 16), V.v.423 (lane 17), V.s.431 (lane 18) and positive control (*V. vulnificus* ATCC 27562) (lane 19).

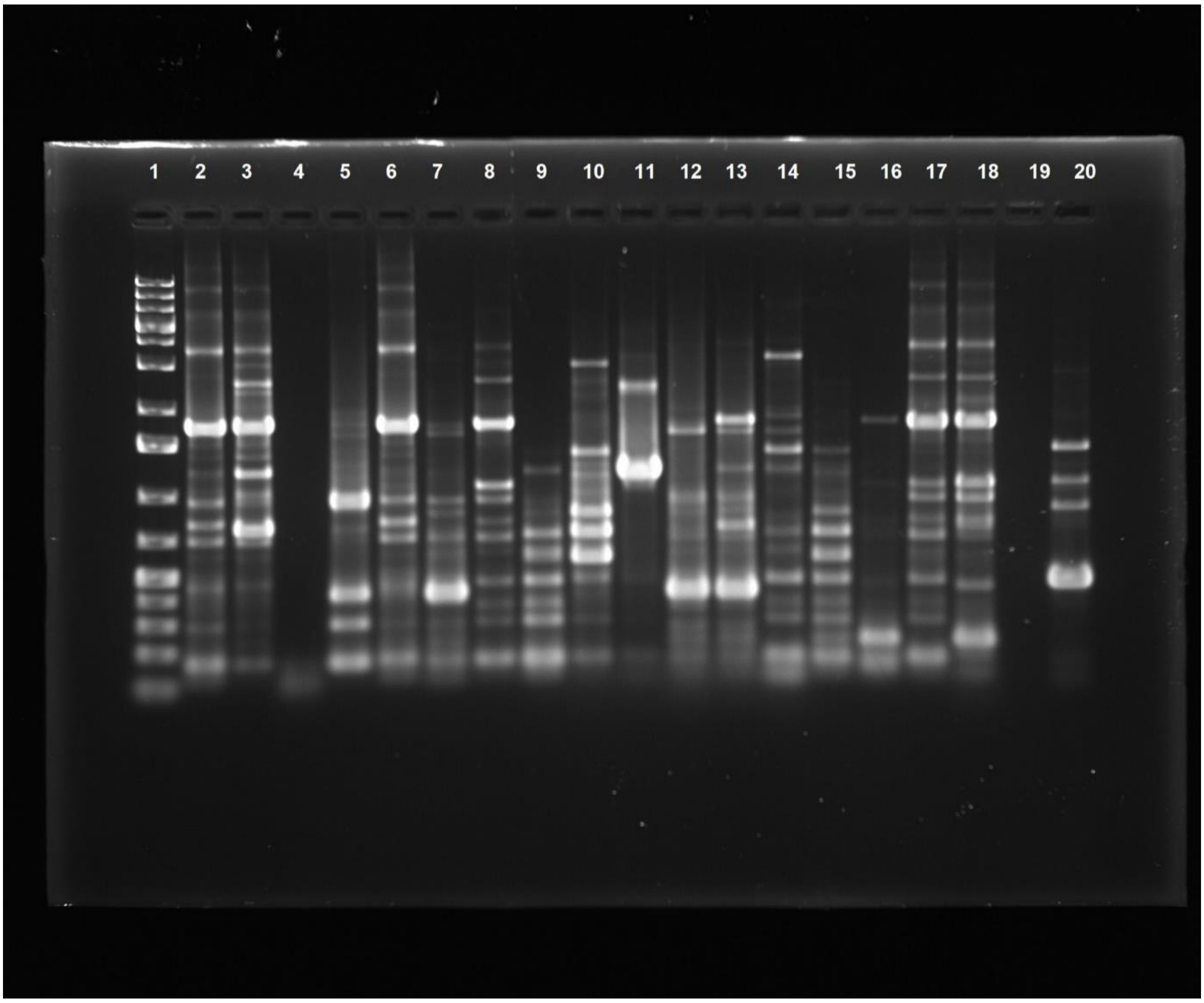

Fig. S5: REP-PCR profiles of the *Vibrio spp*. isolated from treated effluents and upstream and downstream points of receiving rivers. The lanes represent: 1kb molecular weight marker (lane 1), V.v.145 (lane 2), V.v.126 (lane 3), V.v.120 (lane 4), V.v.144 (lane 5), V.v. 145 (lane 6), V.s.102 (lane 7), V.v. 124 (lane 8), V.v. 118 (lane 9), V.v.133 (lane 10), V.v.407 (lane 11), V.v.139 (lane 12), V.v.135 (lane 13), V.v.437 (lane 14), V.v.138 (lane 15), V.v.338 (lane 16), V.v.132 (lane 17), V.v.136(lane 18), negative control (lane 19) and positive control (lane 20).

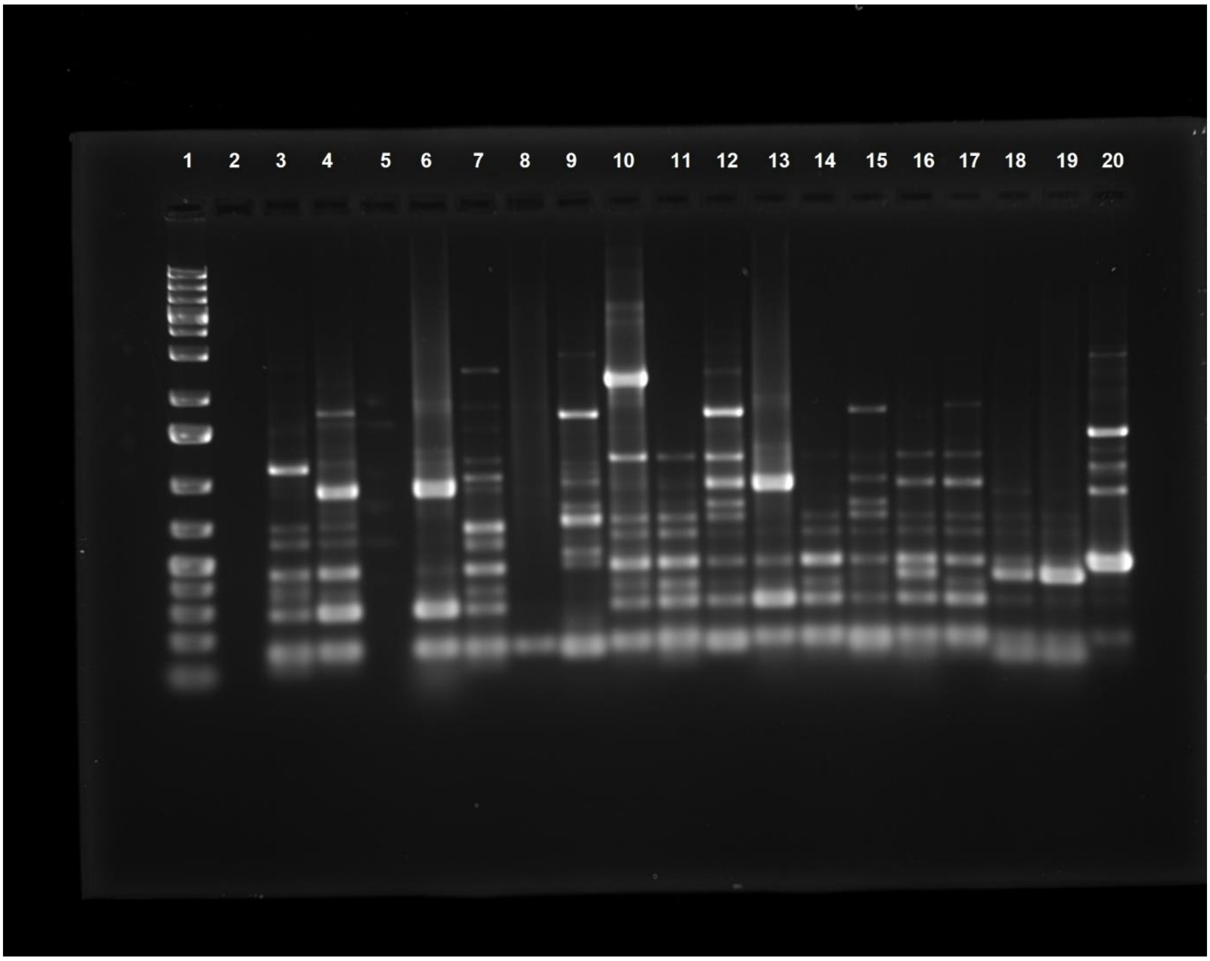

Fig. S6: REP-PCR profiles of the *Vibrio spp*. isolated from treated effluents and upstream and downstream points of receiving rivers. The lanes represent: 1kb molecular weight marker (lane 1), negative control (lane 2), V.v.430 (lane 3), V.v.444 (lane 4), V.v.438 (lane 5), V.v.442 (lane 6), V.v.419 (lane 7), V.v.425 (lane 8), V.v.445 (lane 9), V.v.418 (lane 10), V.v.130 (lane 11), V.v.129 (lane 12), V.v.427 (lane 13), V.s.15 (lane 14), V.v.443(lane 15), V.v.132 (lane 16), V.v.447 (lane 17), V.v.17 (lane 18), V.v.434 (lane 19) and positive control (lane 20).

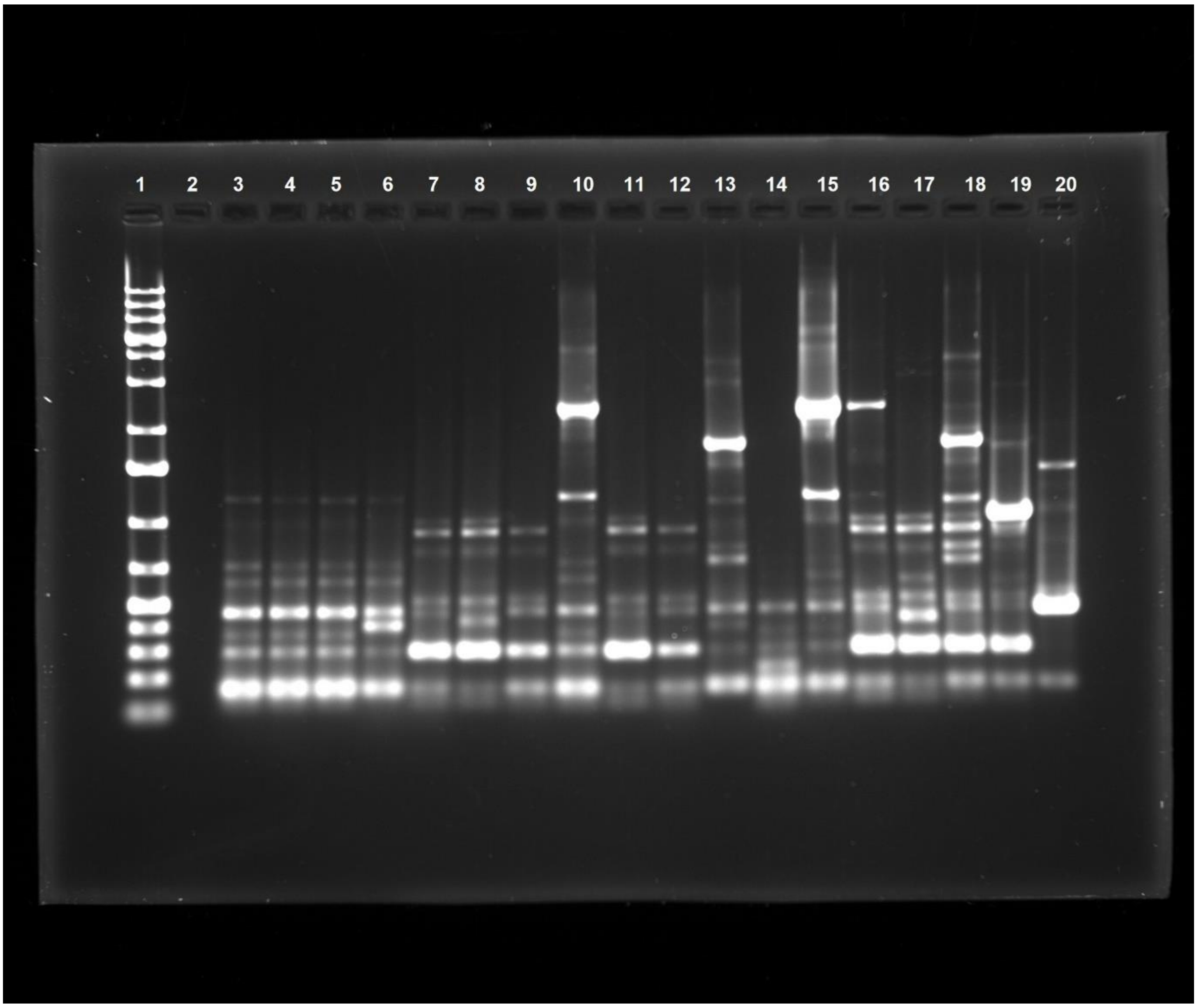

Fig. S7: REP-PCR profiles of the *Vibrio spp*. isolated from treated effluents and upstream and downstream points of receiving rivers. The lanes represent: 1bp molecular weight marker (lane 1), negative control (lane 2), V.v.120 (lane 3), V.v.319 (lane 4), V.v.302 (lane 5), V.v.303 (lane 6), V.v.346 (lane 7), V.v.332 (lane 8), V.v.310 (lane 9), V.v.321 (lane 10), V.v.340 (lane 11), V.v.306 (lane 12), V.v.307 (lane 13), V.v.313 (lane 14), V.v.323 (lane 15), V.v. 320 (lane 16), V.v.324 (lane 17),V.v.344 (lane 18), V.v.322 (lane 19) and positive control (lane 20).

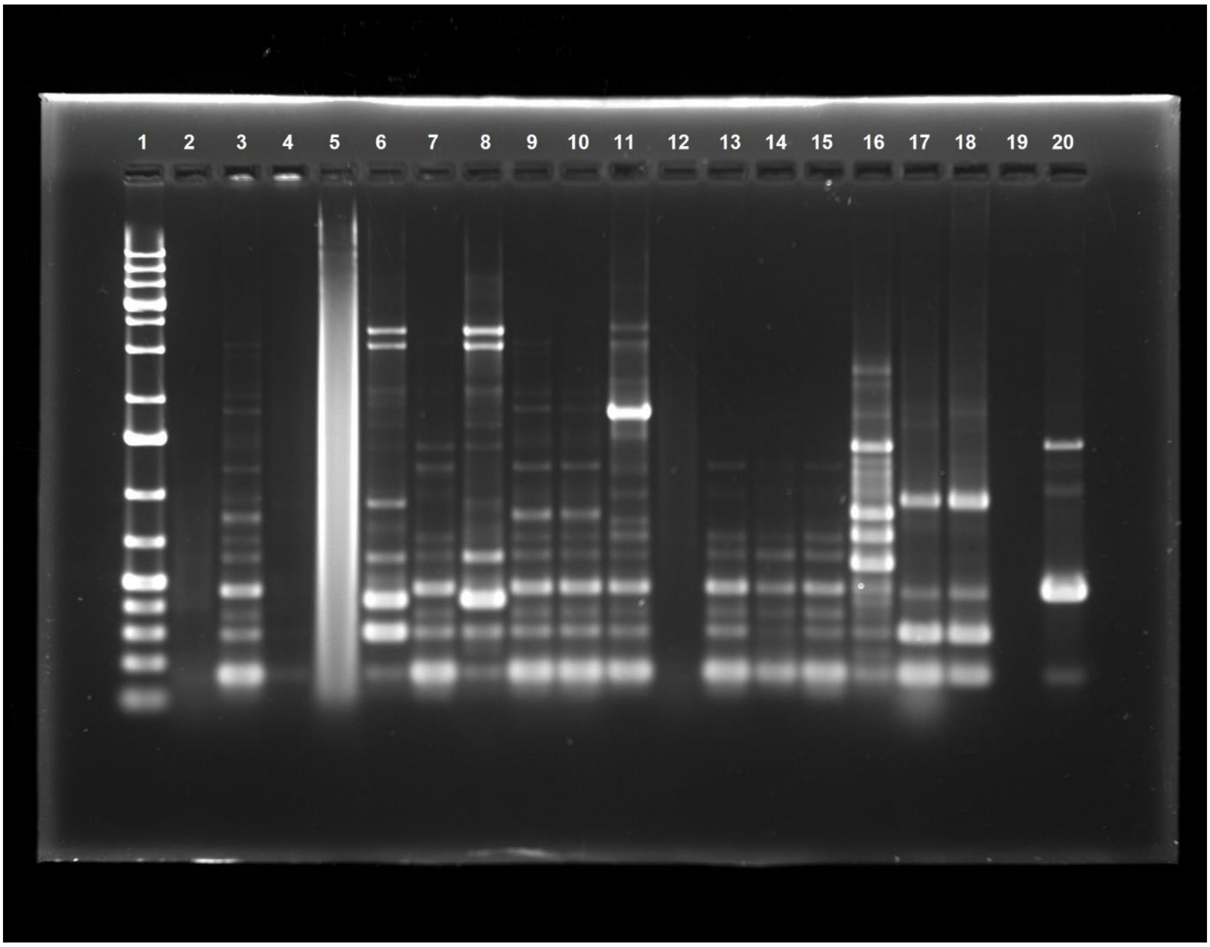

Fig. S8: REP-PCR profiles of the *Vibrio spp*. isolated from treated effluents and upstream and downstream points of receiving rivers. The lanes represent: 1kb molecular weight marker (lane 1), V.v.314 (lane 2), V.v.315 (lane 3), V.v.339 (lane 4), V.v.327 (lane 5), V.v.335 (lane 6), V.v.304(lane 7), V.v.343(lane 8), V.v.330 (lane 9), V.v.316 (lane 10), V.v.317 (lane 11), V.v.311 (lane 12), V.v.301 (lane 13), V.v.341 (lane 14), V.v.326 (lane 15), V.v.302 (lane 16), V.v.435 (lane 17), V.v.448 (lane 18), negative control (lane 19) and positive control (lane 20).

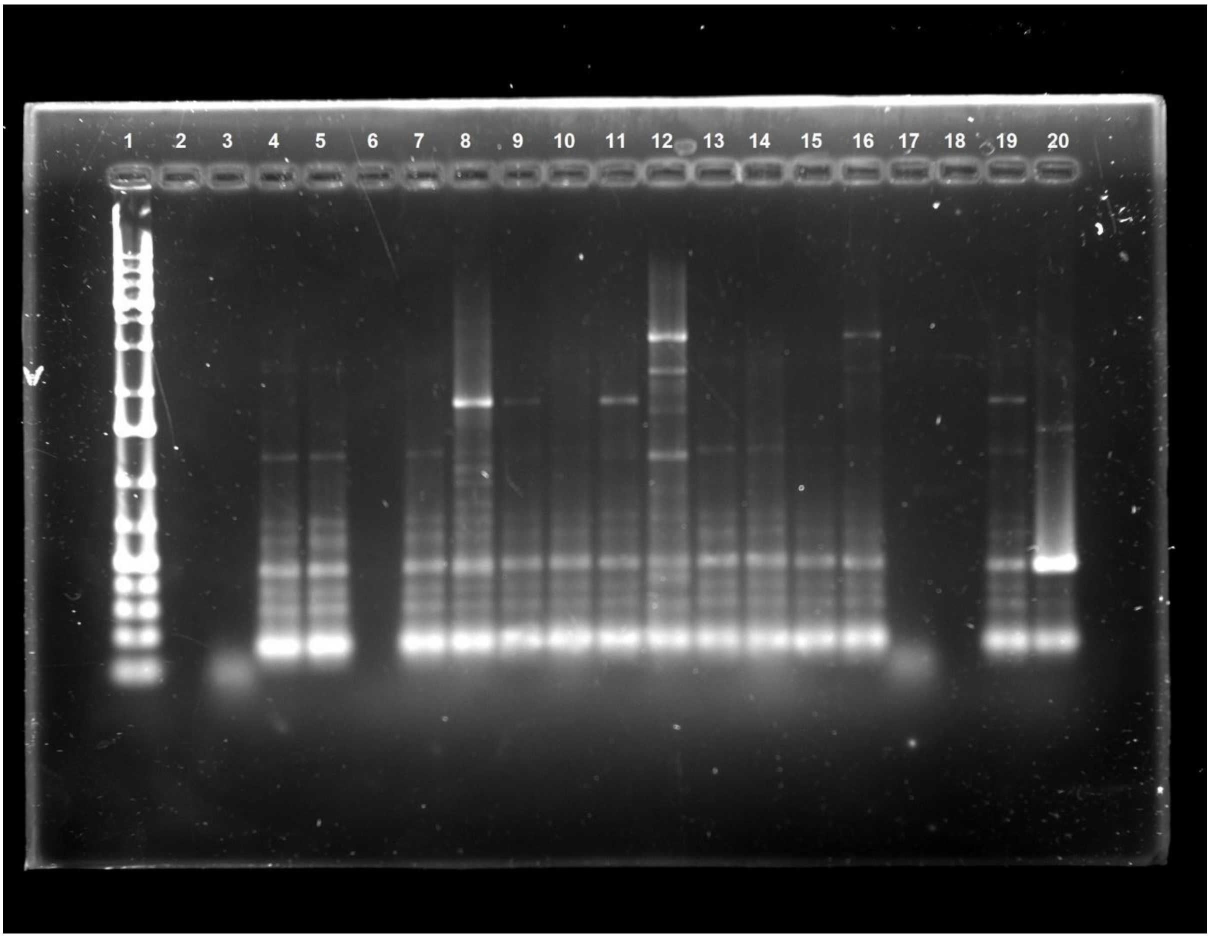

Fig. S9: REP-PCR profiles of the *Vibrio spp*. isolated from treated effluents and upstream and downstream points of receiving rivers. The lanes represent: 1kb molecular weight marker (lane 1), negative control (lane 2), V.s.15 (lane 3), V.v.35(lane 4), V.v.61(lane 5),V.v.47 (lane 6), V.v.62(lane 7), V.v.28 (lane 8), V.v.2 (lane 9), V.s.102 (lane 10), V.v.83 (lane 11), V.v.89(lane 12), V.v.34 (lane 13), V.v.37 (lane 14), V.v.3 (lane 15), V.v.75 (lane 16), V.v.84 (lane 17), V.v.84 (lane 18), V.v.96 (lane 19) and positive control (lane 20).

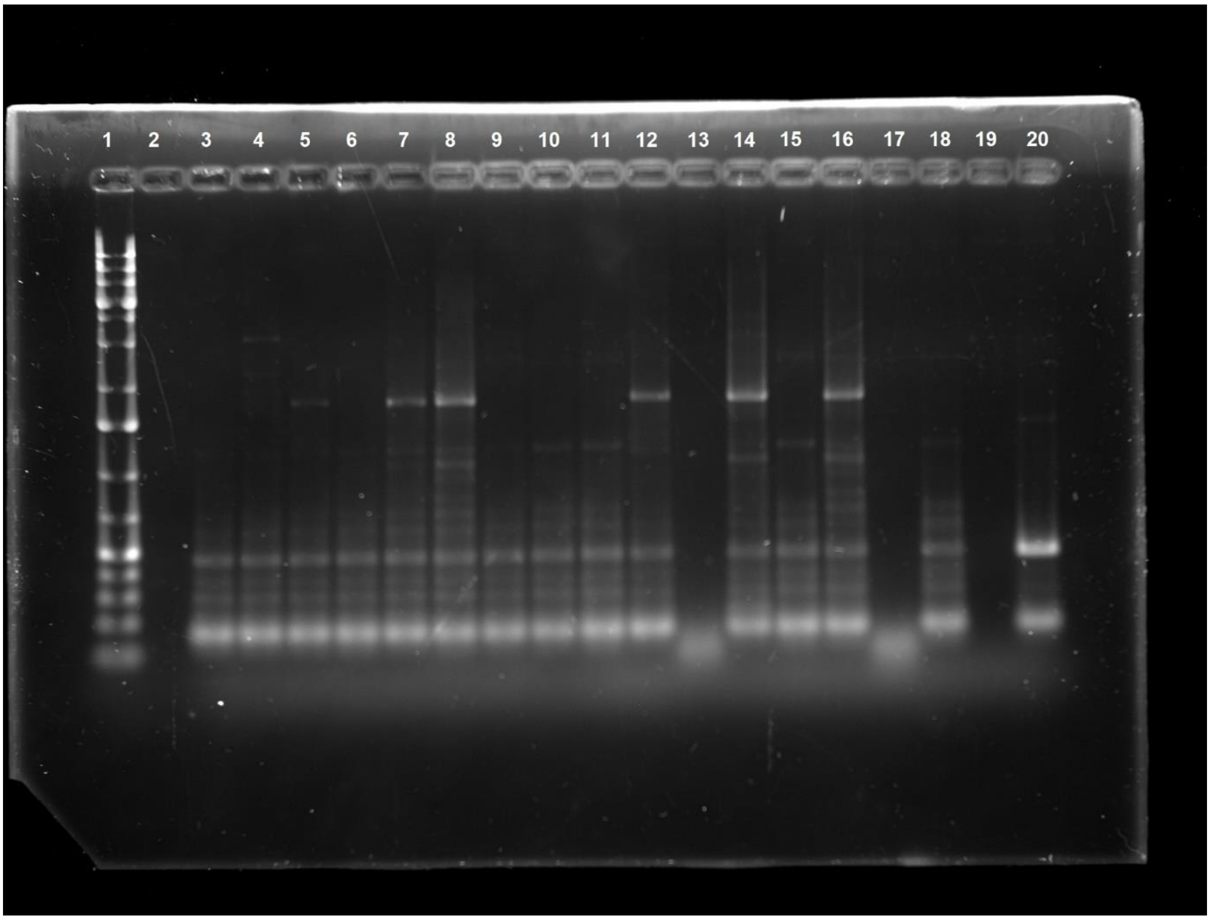

Fig. S10: REP-PCR profiles of the *Vibrio spp*. isolated from treated effluents and upstream and downstream points of receiving rivers. The lanes represent: 1kb molecular weight marker (lane 1), negative control (lane 2), V.v.19 (lane 3), V.v.87 (lane 4), V.v.94 (lane 5), V.v.24 (lane 6), V.v.59(lane 7), V.v.86 (lane 8), V.v.55 (lane 9), V.v.73 (lane 10), V.v.57 (lane 11), V.v.34 (1) (lane 12), V.v.34 (2) (lane 13), V.v.72 (lane 14), V.s.49 (lane 15), V.v.7 (lane 16), V.v.25(lane 17), V.v.51 (lane 18), V.v.93 (lane 19) and positive control (lane 20).

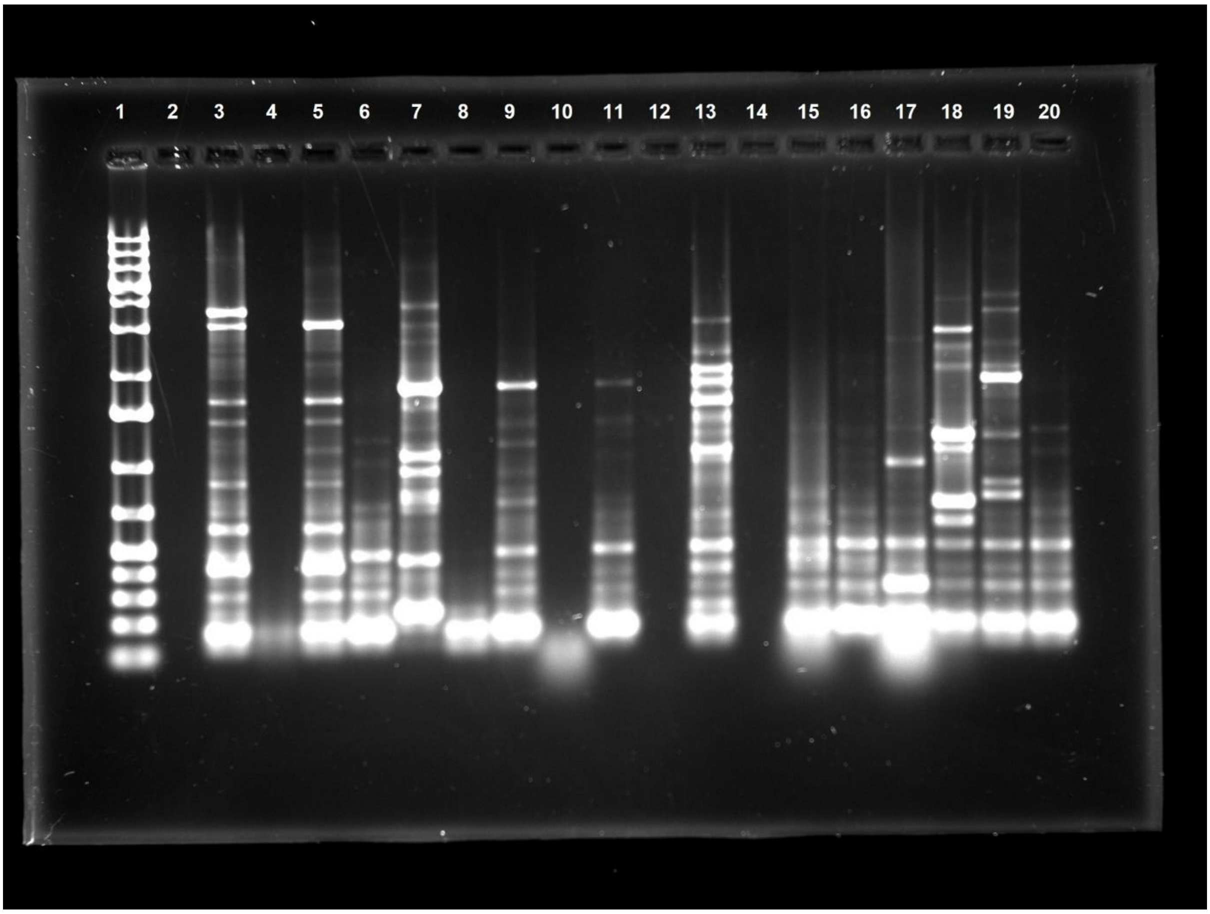

Fig. S11: REP-PCR profiles of the *Vibrio spp*. isolated from treated effluents and upstream and downstream points of receiving rivers. The lanes represent: 1kb molecular weight marker (lane 1), negative control (lane 2), V.v.331 (lane 3), V.v.414 (lane 4), V.a.111 (lane 5), V.a.80 (lane 6), V.a.70 (lane 7), V.a.67 (lane 8), V.a.83 (lane 9), V.a.64(lane 10), V.a.140 (lane 11), V.a.40 (lane 12), V.a.53 (lane 13), V.a.33(lane 14), positive control (*V. alginolyticus* ATCC 17749) (lane 15), V.v.88 (lane 16), V.v.425 (lane 17), V.v.429(lane 18), V.v.342 (lane 19) and positive control (*V. vulnificus* ATCC 27562) (lane 20).

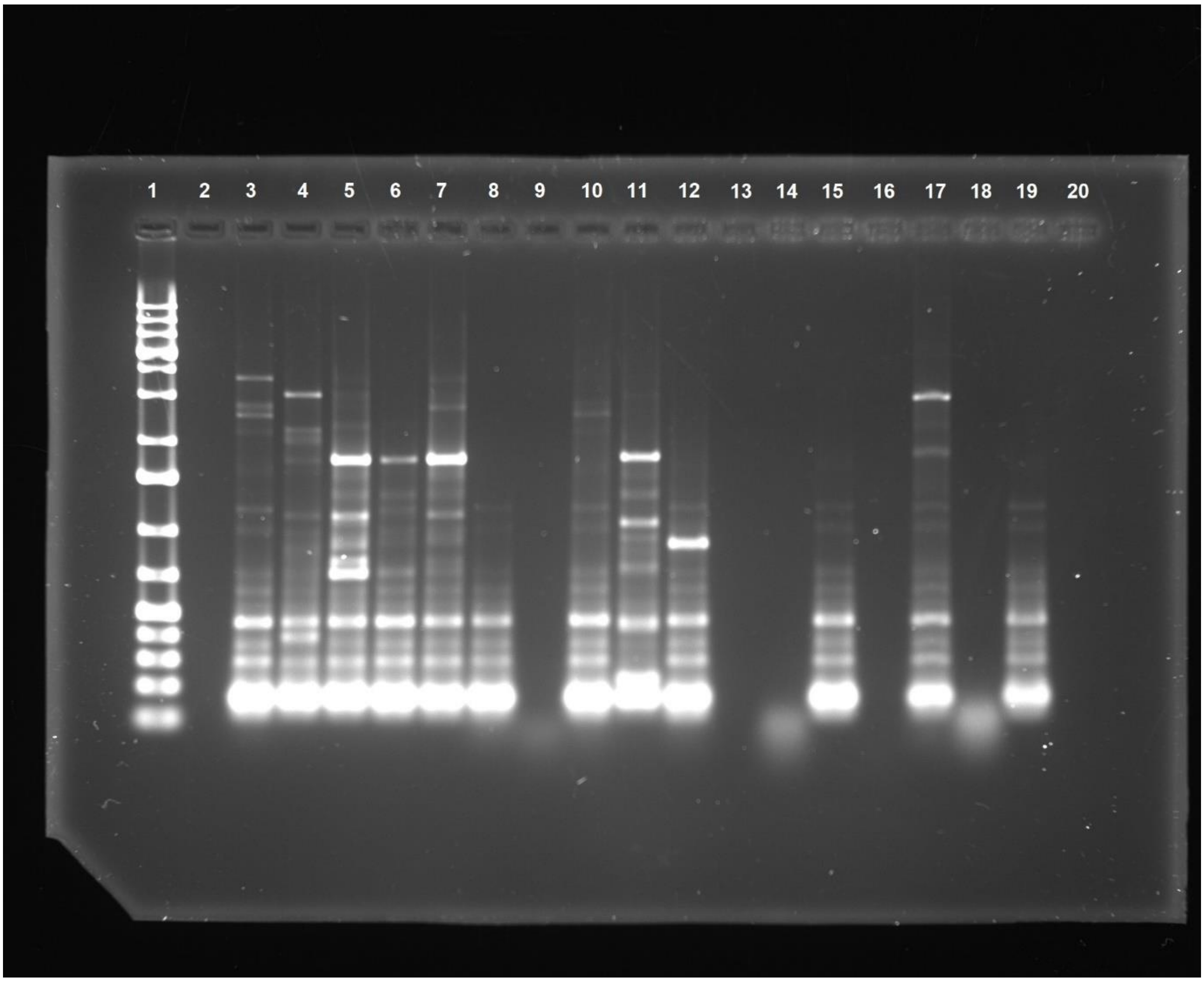

Fig. S12: REP-PCR profiles of the *Vibrio spp*. isolated from treated effluents and upstream and downstream points of receiving rivers. The lanes represent: 1 kb molecular weight marker (lane 1), negative control (lane 2), V.v.77 (lane 3), V.v.311(lane 4), V.v.1 (lane 5), V.v.81 (lane 6), V.v.95 (lane 7), V.s.41 (lane 8), V.v.4 (lane 9), V.v.6 (lane 10), V.v.37 (lane 11), V.v.20 (lane 12), V.s.15(lane 13), V.v.52 (lane 14), V.v.78 (lane 15), V.v.32 (lane 16), V.v.26 (lane 17), V.v.64(lane 18) and positive control (*V. vulnificus* ATCC 27562) (lane 19).

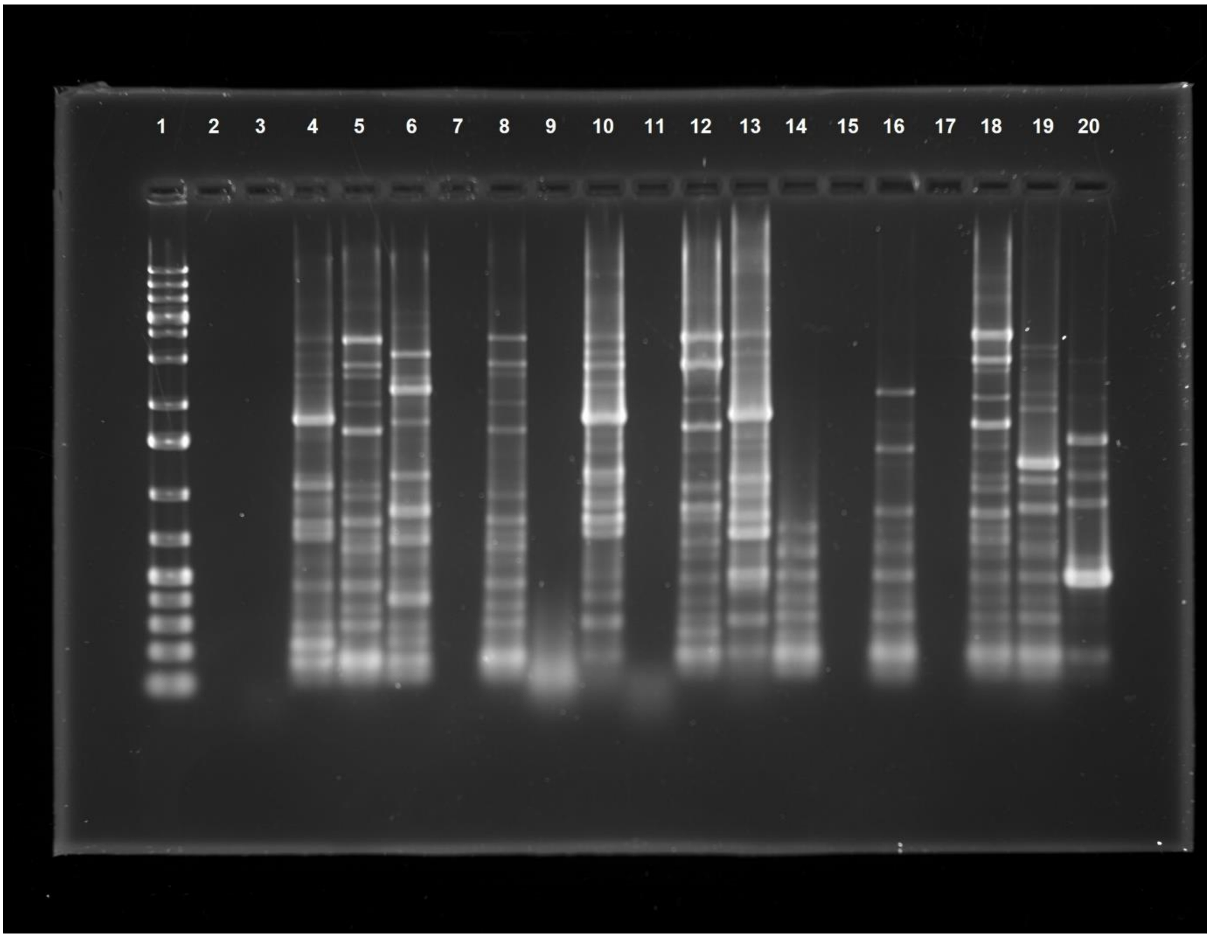

Fig. S13: REP-PCR profiles of the *Vibrio spp*. isolated from treated effluents and upstream and downstream points of receiving rivers. The lanes represent: 1 kb molecular weight marker (lane 1), negative control (lane 2), V.v.29 (lane 3), V.v.48 (lane 4), V.v.58 (lane 5), V.v.87 (lane 6), V.v.21 (lane 7), V.v.4 (lane 8), V.v.51 (lane 9), V.v.82 (lane 10), V.v.29 (lane 11), V.v.45 (lane 12), V.v.85 (lane 13), V.v.137 (lane 14), V.v.17 (lane 15), V.v.50 (lane 16), V.s.18 (lane 17), V.v.60 (lane 18), V.v.43 (lane 19) and positive control (*V. vulnificus* ATCC 27562) (lane 20).

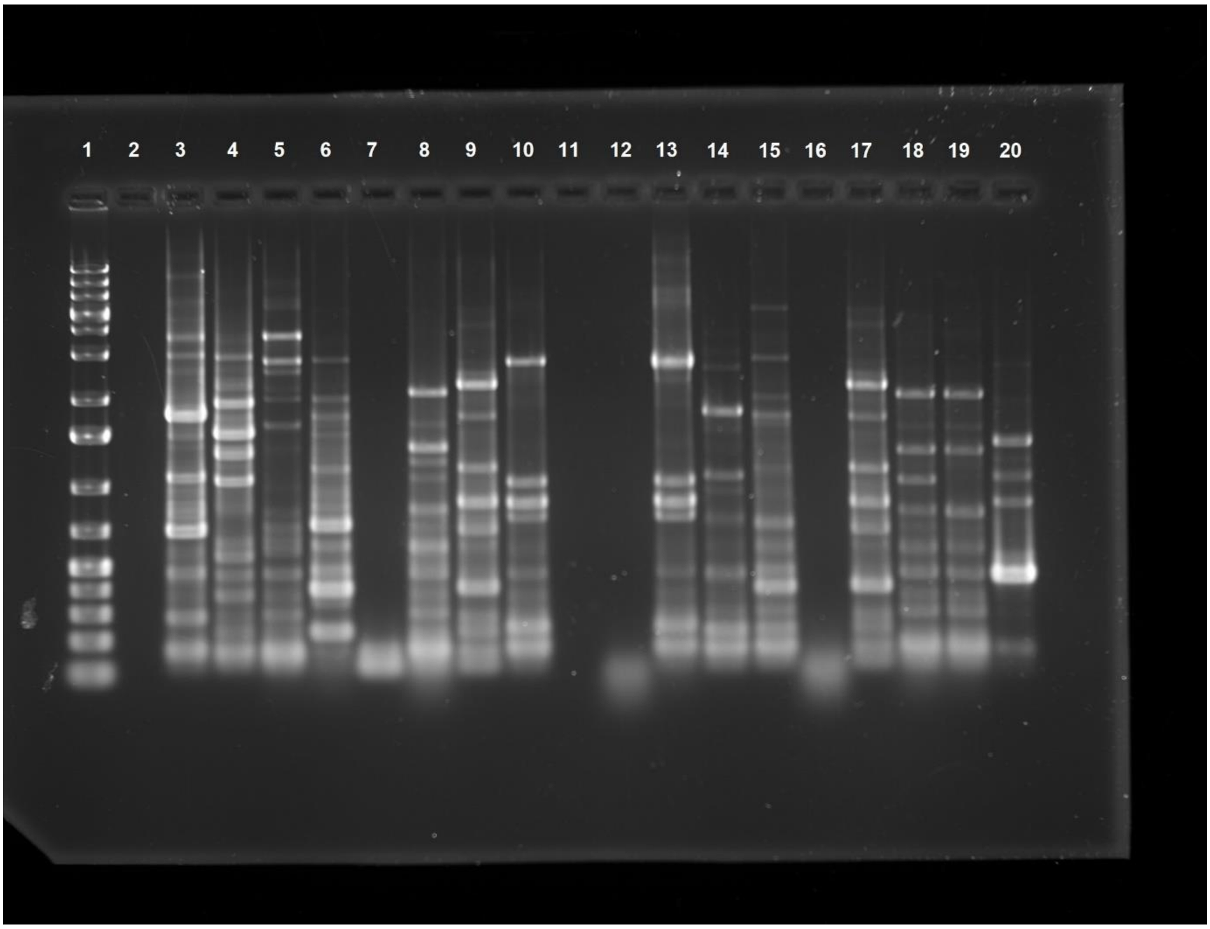

Fig. S14: REP-PCR profiles of the *Vibrio spp*. isolated from treated effluents and upstream and downstream points of receiving rivers. The lanes represent: 1kb molecular weight marker (lane 1), negative control (lane 2),V.v.85 (lane 3), V.v.97 (lane 4), V.v.22 (lane 5), V.v.78 (lane 6), V.v.72 (lane 7), V.v.61 (lane 8), V.v.89 (lane 9), V.v.44 (lane 10), V.v.98 (lane 11), V.v.23 (lane 12), V.v.44 (lane 13), V.v.70 (lane 14), V.v.79 (lane 15), V.v.42 (lane 16), V.v.74 (lane 17), V.v.56 (lane 18), V.v.50 (lane 19) and positive control (*V. vulnificus* ATCC 27562) (lane 20).

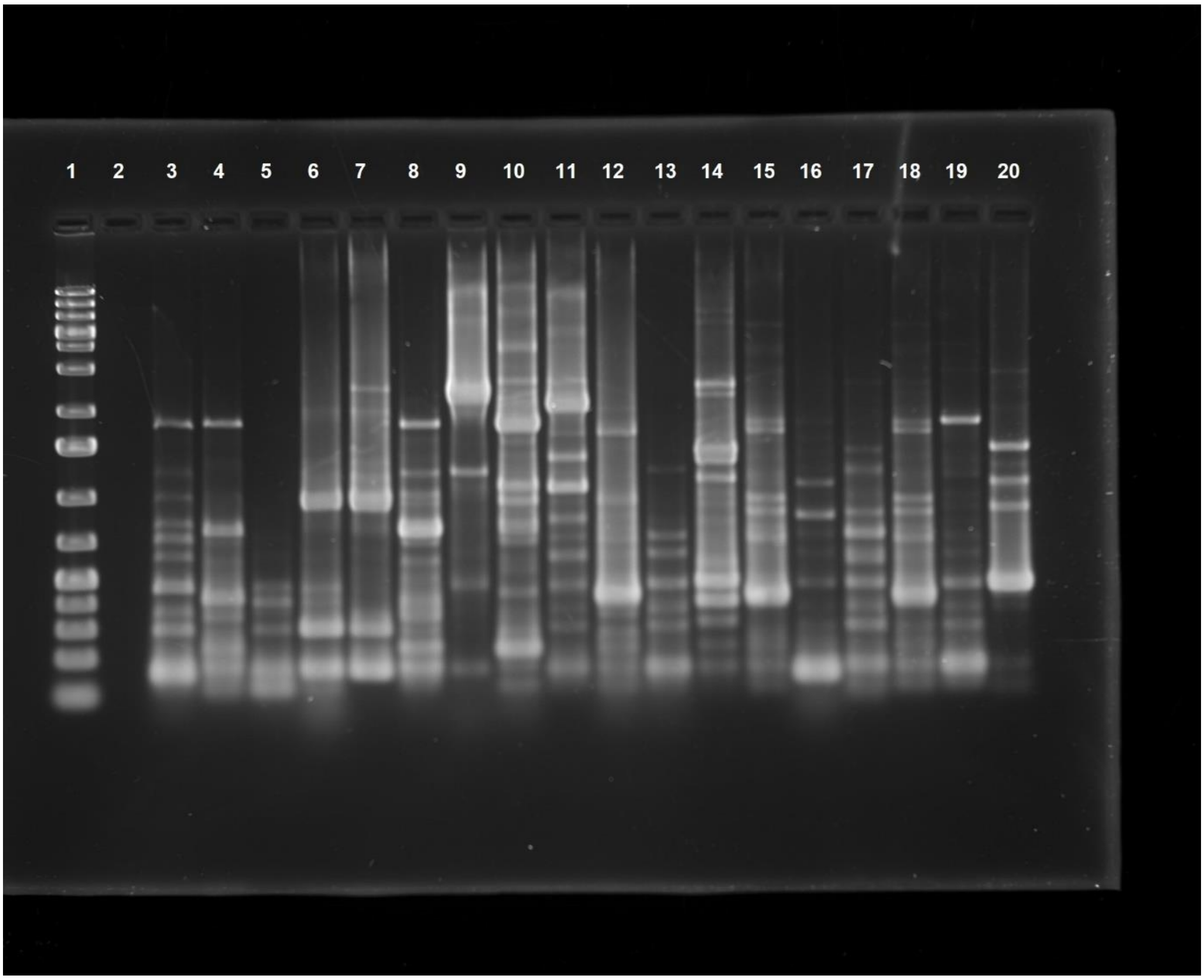

Fig. S15: REP-PCR profiles of the *Vibrio spp*. isolated from treated effluents and upstream and downstream points of receiving rivers. The lanes represent: 1kb molecular weight marker (lane 1), negative control (lane 2), V.v.428 (lane 3), V.v.127 (lane 4), V.v.426 (lane 5), V.v.441 (lane 6), V.v.137 (lane 7), V.v.131 (lane 8), V.v.128 (lane 9), V.v. 130(2) (lane 10), V.v.328 (lane 11), V.v.143 (lane 12), V.v.348 (lane 13), V.v.106 (lane 14), V.v.134 (lane 15), V.v.101 (lane 16), V.v.107 (lane 17), V.v.108 (lane 18), V.v.95 (lane 19) and positive control (*V. vulnificus* ATCC 27562) (lane 20).

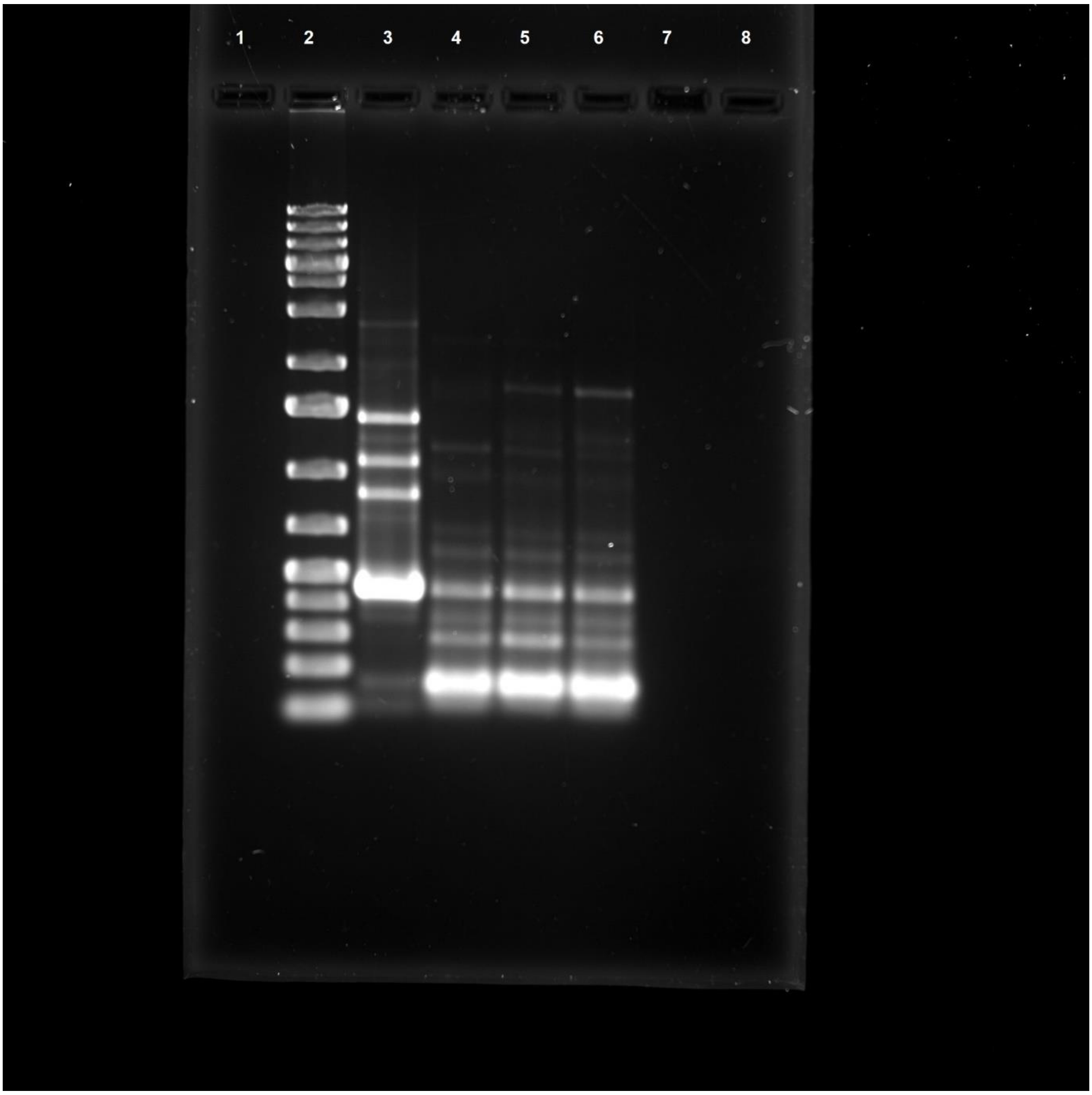

Fig. S16: REP-PCR profiles of the *Vibrio spp*. isolated from treated effluents and upstream and downstream points of receiving rivers. The lanes represent: 1kb molecular weight marker (lane 1), positive control (*V. vulnificus*) (lane 2), V.v.56 (lane 3), V.v.7 (lane 4) and V.v.28 (lane 5).

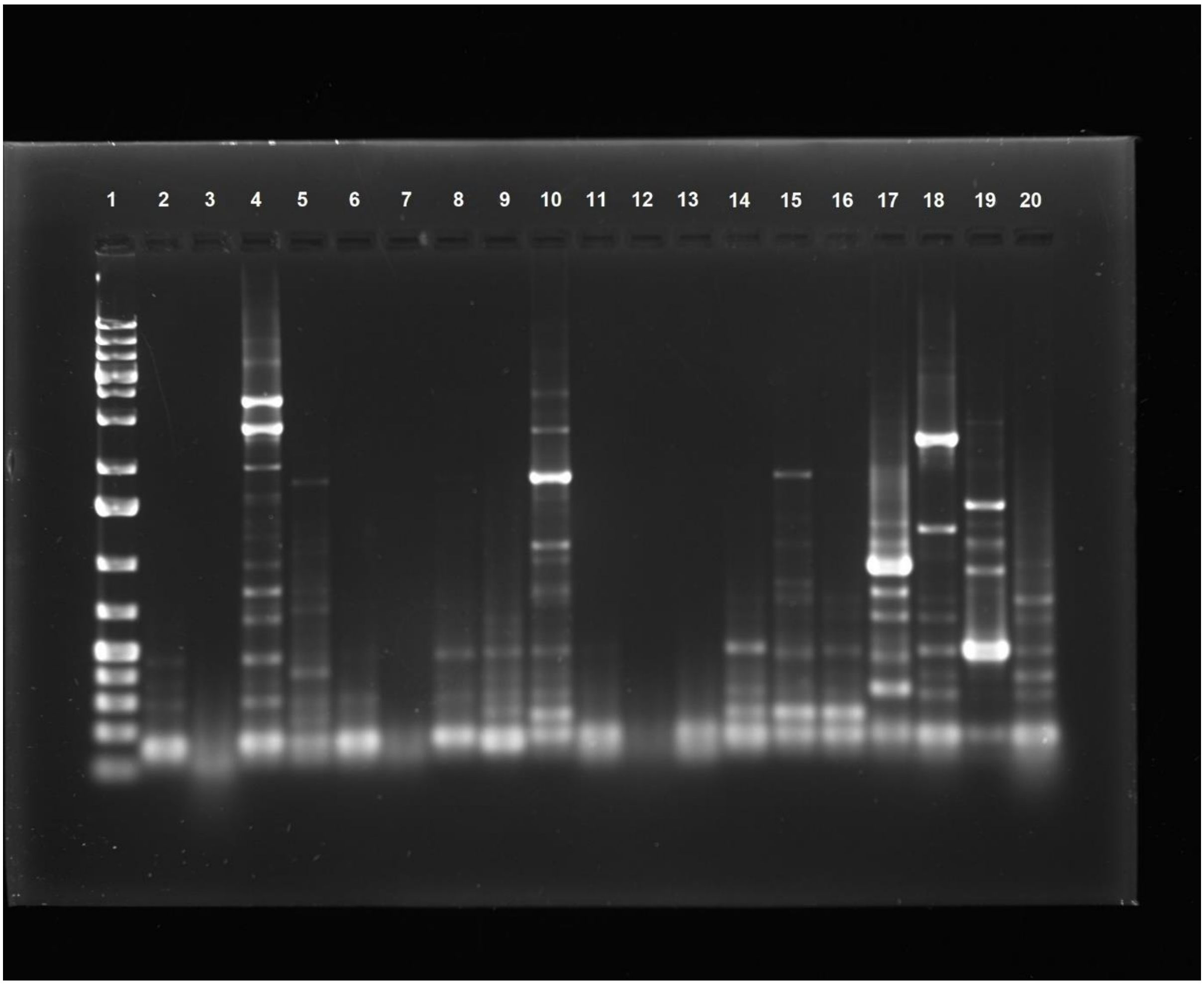

Fig. S17: REP-PCR profiles of the *Vibrio spp*. isolated from treated effluents and upstream and downstream points of receiving rivers. The lanes represent: 1kb molecular weight marker (lane 1), V.v.4 (lane 2), V.v.17 (lane 3), V.v.34 (lane 4), V.v.51 (lane 5), V.v.93 (lane 6), V.v.84 (lane 7), V.v.84 (lane 8), V.v.29 (lane 9), V.a.67 (lane 10), V.a.33 (lane 11), V.a.15 (lane 12), V.v.34 (lane 13), V.v.25 (lane 14), V.a.64 (lane 15), V.a.64 (lane 16), V.v.20 (lane 17), V.v.414 (lane 18), positive control (*V. vulnificus* ATCC 27562) (lane 19) and positive control (*V. alginolyticus* 17749) (lane 20).

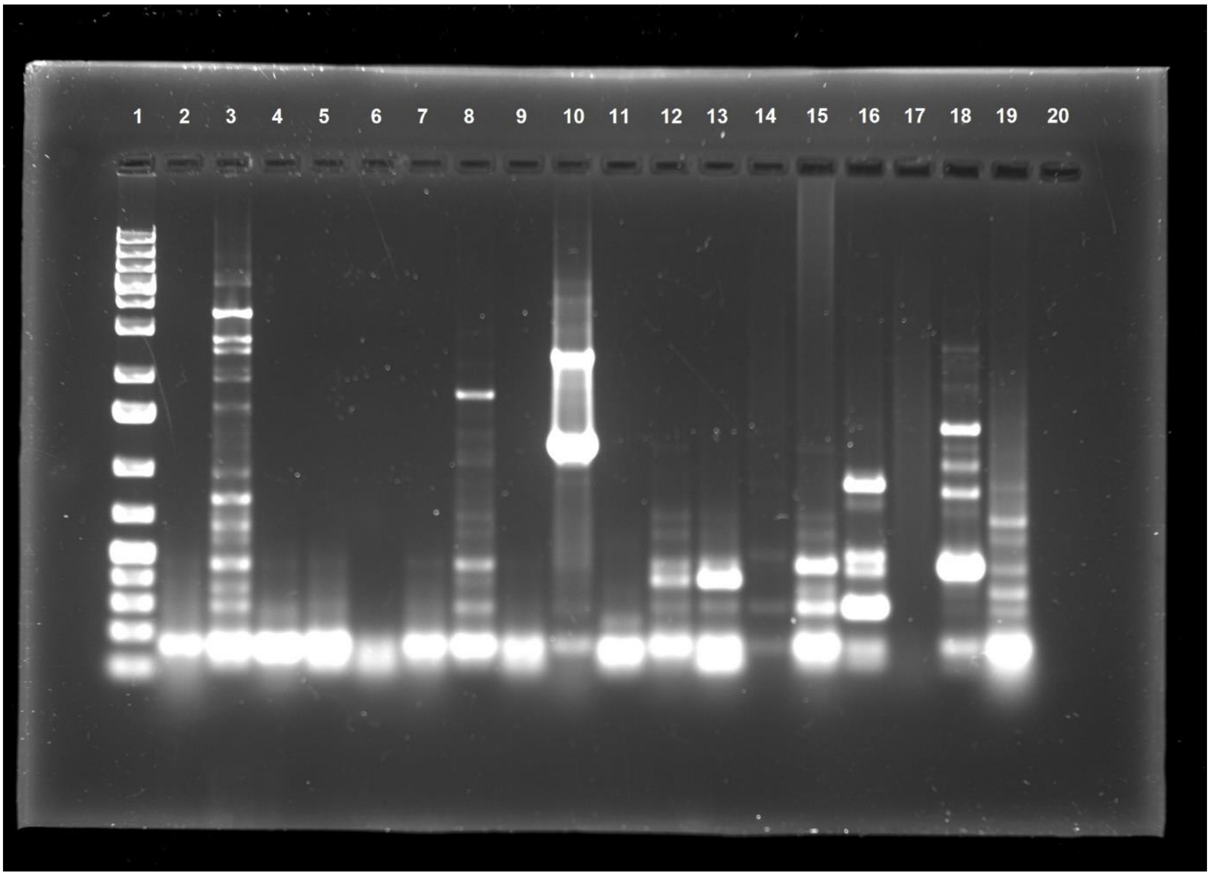

Fig. S18: REP-PCR profiles of the *Vibrio spp*. isolated from treated effluents and upstream and downstream points of receiving rivers. The lanes represent: 1kb molecular weight marker (lane 1), V.v.21 (lane 2), V.v.4 (lane 3), V.v.32 (lane 4), V.v.47 (lane 5), V.v.18 (lane 6), V.a.40 (lane 7), V.v.51 (lane 8), V.v.120 (lane 9), V.v.411 (lane 10), V.v.29 (lane 11), V.v.314 (lane 12), V.v.413 (lane 13), V.v.339 (lane 14), V.v.438 (lane 15), V.v.327 (lane 16), V.v.311 (lane 17), positive control (*V. vulnificus* ATCC 27562) (lane 18) and positive control (*V. alginolyticus* 17749) (lane 19).
